# RCN-MAMBA: Research on Pedestrian-Future-Trajectory-Prediction Methods for Occlusion Scenarios

**DOI:** 10.3390/s26185926

**Published:** 2026-09-19

**Authors:** Sijie Yang, Zhaoyu Li, Guoyu Lin, Rongrong Ni, Biao Yang

**Affiliations:** 1 Wang Zheng Microelectronics College, Changzhou University, Changzhou 213164, China; s24060809002@smail.cczu.edu.cn (S.Y.); nirongrong@cczu.edu.cn (R.N.); 2 School of Overseas Education, Changzhou University, Changzhou 213164, China; 2400460313@smail.cczu.edu.cn; 3 Department of Intelligent Equipment, Changzhou University of Information Technology, Changzhou 213164, China; linguoyu@czcit.edu.cn

**Keywords:** pedestrian-future-trajectory prediction, occluded-trajectory completion, physics-aware residual completion, explicit motion-state encoding, multi-scale temporal convolution

## Abstract

Pedestrian-future-trajectory prediction is a critical task in intelligent driving, traffic-scene understanding, and active safety decision-making. In real-world road environments, pedestrians’ historical trajectories are frequently affected by occlusions from vehicles, other pedestrians, road infrastructure, and detector-missed detections, leading to missing values in the historical-observation sequence. Understanding and handling missing values in pedestrian-observation sequences is essential for improving the performance of prediction models; however, existing research has rarely considered this realistic scenario. This paper proposes RCN-MAMBA, a pedestrian-future-trajectory-prediction method for occlusion scenarios. The overall pipeline follows a de-occlusion completion first, future-trajectory-prediction second paradigm. Specifically, in the de-occlusion stage, based on linear interpolation for trajectory completion, a BiLSTM Residual-Correction Network is employed to further refine the occluded trajectory. This paper further proposes the C_MAMBA prediction architecture: first, explicit motion-state encoding is introduced to inject position, velocity, and acceleration information explicitly into the trajectory representation; then, multi-scale temporal convolution is used to extract local motion patterns across different time ranges; finally, a Temporal Bi-Mamba module is introduced to model long-term temporal dependencies in the completed trajectory from both forward and backward temporal directions. RCN-MAMBA achieves competitive performance on the JAAD dataset. Qualitative results show that its predicted future trajectories are consistent with actual motion trends. Quantitative analysis is conducted under six whole-frame random occlusion ratios: 5%, 10%, 15%, 20%, 30%, and 40%. RCN-MAMBA achieves optimal results under all these occlusion ratios. The paired-t test for each trajectory further confirms that the performance advantage of our proposed method over all baseline methods is statistically significant, demonstrating that the proposed method possesses good prediction accuracy and robustness under varying occlusion intensities.

## 1. Introduction

With the rapid development of intelligent driving technology and advanced driver-assistance systems, autonomous vehicles’ perception and understanding of surrounding traffic environments are undergoing a profound transformation from perceiving the present to predicting the future. In complex dynamic traffic scenes, pedestrians, as the most vulnerable traffic participants, exhibit highly random, nonlinear, and intention-uncertain motion behaviors. Accurately predicting pedestrians’ position changes over a future time period not only helps intelligent vehicles identify potential collision risks in advance and plan safe driving paths, but is also a key prerequisite for achieving vehicle–road collaboration and human–machine co-driving. Therefore, pedestrian-future-trajectory prediction has become a core task in the autonomous-driving perception–prediction–decision pipeline, receiving widespread attention from both academia and industry.

Early pedestrian-trajectory-prediction methods were mainly based on physical-motion models and statistical methods, establishing motion-state equations through constant-velocity models, social-force models, or Kalman filtering to describe pedestrians’ motion patterns. These methods are structurally simple and highly interpretable, but often fall short in the face of complex nonlinear behaviors in real traffic scenes. With the development of deep learning, data-driven trajectory-prediction methods have gradually become mainstream. Social-LSTM [1] was the first to utilize recurrent neural networks to learn historical-trajectory temporal features and model pedestrian interactions through a social pooling mechanism; Social-GAN [2] further introduced generative adversarial networks to learn future-trajectory distributions, improving the diversity of prediction results. Subsequently, graph neural network methods explicitly modeled multi-agent interaction processes by constructing relationship graphs between pedestrians and surrounding traffic participants. Yang et al. [3] proposed a multi-task learning network that models interactions among traffic participants through a collision-aware graph transformer, simultaneously considering motion consistency and collision safety in trajectory prediction, and further enriching the application paradigm of graph neural networks in trajectory prediction.

In recent years, the model-architecture evolution in the trajectory-prediction field has shown two noteworthy trends. On the one hand, Transformer structures have continued to leverage their advantage in long-distance dependency modeling through self-attention mechanisms: HPnet [4] achieves dynamic trajectory prediction through historical prediction attention; ASTRA [5] combines scene awareness with Transformer to enhance environmental understanding; and SIAT [6] further exploits group motion patterns through a socially interactive-aware Transformer. Ni et al. [7] proposed an adaptive progressive Transformer for trajectory prediction under fine-grained trajectory-scene interaction constraints, enhancing the model’s perception capability of complex environmental semantics. On the other hand, state-space models (SSM) represented by Mamba offer new possibilities for temporal modeling. Trajectory Mamba (CVPR 2025) [8] introduces selective state-space mechanisms into trajectory prediction, enhancing long-range temporal dependency modeling while maintaining linear computational complexity; iDMaTraj [9] explores the fusion path of Mamba and diffusion models; and TrajMamba [10] further adopts an ego-motion perspective, utilizing Mamba for egocentric trajectory-prediction tasks. Xu et al. [11] explored the fusion of language models and reinforcement learning in trajectory prediction, proposing the W2W method, which provides a new technical path for trajectory generation based on high-level semantic understanding. These works indicate that Mamba structures possess both efficiency and accuracy advantages when processing long-sequence trajectory data, providing important inspiration for the model design in this paper.

However, most of the above studies implicitly assume that historical-observation trajectories are complete without missing values, ignoring the ubiquitous occlusion problem in real autonomous-driving scenarios. Due to vehicle occlusion, mutual occlusion among pedestrians, road-facility occlusion, and target-detection-algorithm missed detections, pedestrians’ historical trajectories often exhibit partial unobservability, resulting in broken trajectory sequences. Occlusion destroys the continuity of historical trajectories, preventing prediction models from fully obtaining pedestrians’ motion directions, velocity changes, and potential motion trends. If trajectories with missing information are directly fed into prediction models, the models may misinterpret missing regions as abnormal motion patterns, thereby severely affecting the accuracy and reliability of future-trajectory prediction. Therefore, under occlusion conditions, merely improving the modeling capability of future-trajectory-prediction models cannot completely solve the problem; it is also necessary to first recover the missing historical-motion information, which is of great significance for enhancing the robustness of prediction models in real scenarios.

To address the trajectory-missing problem, some studies have attempted to use interpolation, filtering, and deep learning methods for trajectory completion. Traditional interpolation methods utilize visible trajectory points before and after the missing region to recover continuous trajectories, with the advantages of simple computation and high stability. However, such methods usually rely on local continuity assumptions and struggle to describe complex motion changes during pedestrian acceleration, deceleration, and turning, easily producing over-smoothed results. In recent years, deep learning-based time-series-completion methods such as BRITS [12] and SAITS [13] have been able to utilize richer contextual information to recover missing data, but most methods directly learn the mapping relationship for missing positions, lacking explicit constraints on motion laws such as velocity and acceleration, and still exhibit recovery deviations under complex motion conditions. Furthermore, existing work mostly treats trajectory completion and future prediction as two independent tasks, failing to fully consider the friendliness of recovered trajectories to downstream prediction tasks, resulting in completion results that may be numerically close to real trajectories but not necessarily effective in improving prediction performance.

To address the above challenges, this paper proposes RCN-MAMBA, a two-stage pedestrian-future-trajectory-prediction method for occlusion scenarios following a de-occlusion completion first, future-trajectory-prediction second paradigm. In the de-occlusion stage, this paper first employs mean interpolation for preliminary recovery of missing trajectories, providing stable initial values for subsequent refinement; on this basis, a Physics-Aware Residual Completion Network is proposed, which constructs motion features including position, velocity, acceleration, and occlusion position information, and utilizes a BiLSTM residual-correction network to learn dynamic changes in occluded regions, recovering more kinematically consistent complete trajectories through integration. In the future-trajectory-prediction stage, this paper further proposes the C_MAMBA prediction architecture: first, Explicit Motion-State Encoding (EME) is introduced to explicitly inject position, velocity, and acceleration information into the trajectory representation, enhancing the model’s perception capability of dynamic motion information; then, Multi-Scale Temporal Convolution (MTC) is used to extract local motion patterns across different time ranges; finally, a Temporal Bi-Mamba module is introduced to model long-term temporal dependencies in the completed trajectory from both forward and backward temporal directions, improving the model’s understanding capability of historical-motion-state changes and overall trends.

The main contributions of this paper include the following three aspects:

First, addressing the problem of missing information in historical trajectories caused by occlusion in real traffic environments, this paper studies the pedestrian-future-trajectory-prediction task under occlusion conditions and proposes a two-stage prediction framework of trajectory completion–future prediction.

Second, a trajectory de-occlusion method based on interpolation initialization and physics-aware residual correction is proposed. This method utilizes interpolation results to provide stable initial trajectories and learns dynamic changes in occluded regions through a motion-feature-driven residual network, achieving more accurate trajectory recovery.

Third, a MAMBA future-trajectory-prediction architecture is proposed. By fusing explicit motion-state encoding, multi-scale temporal convolution, and the Temporal Bi-Mamba module, the modeling capability for dynamic motion information, multi-scale temporal patterns, and long-term temporal dependency relationships is improved.

Experimental results show that RCN-MAMBA achieves competitive performance on both JAAD and PIE datasets. Under occlusion-free conditions, the proposed method achieves ADE0.5s/1.0s/1.5s of 25/57/112 on the JAAD dataset and 13/28/52 on the PIE dataset, outperforming existing representative methods. Under occlusion scenarios, this paper tests six whole-frame random occlusion ratios: 5%, 10%, 15%, 20%, 30%, and 40%. RCN-MAMBA achieves optimal results under all occlusion ratios. Taking 40% occlusion on the JAAD dataset as an example, the model achieves ADE 45.7/97.4/188.6, FDE 391.5, C-ADE 150.8, and C-FDE 358.6, representing reductions of approximately 47.1%, 47.3%, 44.9%, 46.5%, 46.6%, and 40.6% compared to the baseline, respectively, fully verifying the prediction accuracy and robustness of the proposed method under different occlusion intensities.

The remainder of this paper is organized as follows: Section 2 reviews related work on pedestrian trajectory prediction, occluded-trajectory completion, and temporal modeling; Section 3 details the proposed RCN-MAMBA method, including problem definition, the physics-aware residual completion de-occlusion module, and the MAMBA future-trajectory-prediction module; Section 4 reports experimental settings, quantitative and qualitative analyses, and ablation study results; and Section 5 summarizes the full paper and discusses future research directions.

## 2. Related Work

### 2.1. Pedestrian-Future-Trajectory Prediction

Pedestrian-future-trajectory prediction aims to infer future positions based on historical observations of trajectories and is a key link in the autonomous-driving perception–prediction–decision pipeline. In recent years, the methodology in this field has undergone profound transformations from deterministic modeling to generative modeling, and from unimodal output to multimodal probability distributions.

Trajectory prediction based on Generative Models has become one of the mainstream paradigms in current research. Trajectron++ [14] combines scene graphs with pedestrian kinematic constraints based on a conditional variational autoencoder, enabling the generation of multimodal trajectory distributions. Diffusion Models, with their ability to characterize complex multimodal distributions, have demonstrated significant advantages in trajectory prediction. The MID [15] proposed by Gu et al. was the first to introduce the diffusion process into pedestrian trajectory prediction, generating future trajectories by simulating the inverse process from uncertainty to determinacy. Subsequently, Mao et al. [16] proposed LED, which accelerates denoising sampling through a learnable leapfrog initializer and Wang et al. [17] c proposed C2F-TP, adopting a coarse-to-fine denoising framework to explicitly model prediction uncertainty. However, the above methods mostly focus on generating trajectory distributions, with insufficient explicit modeling of pedestrian-motion intentions. To this end, recent research has begun to embed intention information into the diffusion process: IAD [18] proposed an intention-aware diffusion framework, modeling short-term intentions through residual polar coordinate representations and estimating multimodal long-term intentions with a tokenized endpoint predictor; Fu et al. [19] proposed MoFlow, introducing Flow Matching into trajectory prediction to achieve efficient inference through single-step generation; and Xing et al. [20] proposed GoalFlow, which drives flow matching for multimodal trajectory generation conditioned on goals. Furthermore, Wang et al. [9] proposed iDMaTraj, combining Mamba with diffusion models to improve long-sequence modeling efficiency while maintaining generation quality. These works indicate that generative models are evolving from pure distribution fitting to joint intention-distribution modeling.

Trajectory prediction based on Transformer continues to deepen in terms of global dependency modeling. The Trajectory Unified Transformer proposed by Shi et al. [21] achieves joint modeling of historical and future trajectories through a unified encoder-decoder architecture. Recently, HPnet [4] introduced historical-prediction attention mechanisms to dynamically utilize prediction information from past moments to assist current decisions; ASTRA [5] combines scene semantics with Transformer to enhance perception of static environments; and SIAT [6] explicitly exploits group-motion patterns through a socially interactive-aware Transformer. The adaptive progressive Transformer proposed by Ni et al. [7] further optimizes the prediction process under fine-grained trajectory-scene interaction constraints, improving the model’s perception accuracy of complex environmental semantics through progressive feature refinement. Additionally, Social-Transmotion [22] explores the application of prompting in trajectory prediction, allowing generation behavior to be controlled through text prompts, opening new directions for controllable prediction. These advances indicate that Transformer architectures are evolving from general sequence modeling tools toward multi-dimensional fusion of scene, interaction, and intention.

Graph Neural Networks and Interaction Modeling: Social-STGCNN [23] was the first to introduce graph convolutional networks into pedestrian trajectory prediction, efficiently modeling group motion through a spatiotemporal interaction graph constructed with distance kernels and temporal convolutions. SocialCircle [24] proposes an angle-based social interaction representation method that explicitly encodes the azimuth relationships between pedestrians; Kim et al. [25] capture complex interactions among multiple agents through higher-order relational reasoning; and Lin et al. [26] proposed GigaTraj, achieving long-term trajectory prediction for hundreds of pedestrians in gigapixel complex scenes. In ego-centric prediction tasks, Rasouli [27] proposed a scale- and motion-aware model, emphasizing the importance of handling scale variations in the egocentric coordinate system; RealTraj [28] systematically investigated distribution shift and data quality issues in real-world trajectory prediction. DeMo [29] decouples motion prediction into two sub-problems of directional intention and dynamic state, improving the model’s understanding of fine-grained motion patterns. Yang et al. [3] proposed a multi-task learning network introducing a collision-aware graph transformer into trajectory prediction, explicitly incorporating collision risk constraints into graph-structured interaction modeling, and making prediction results more advantageous in safety perception. Furthermore, pedestrian-trajectory data in real traffic scenes often exhibits a long-tail distribution, where a few common motion patterns dominate while a large number of rare patterns are difficult to learn fully. In terms of rapid model adaptation, Yang et al. [30] proposed the Meta-IRLSOT++ method based on meta inverse reinforcement learning, achieving fast adaptive trajectory-prediction networks and providing an effective approach for handling scene distribution changes and Yang et al. [31] further explored an online multi-source transfer learning framework, improving model generalization in new scenarios through cross-domain knowledge transfer.

Notably, the above studies mostly assume that historical observations of trajectories are complete without missing values. However, in real autonomous-driving scenarios, the problem of trajectory breakage caused by detector-missed detections and occlusion is ubiquitous. MS-TIP [32] was the first to systematically study the joint modeling of trajectory completion and prediction at ICML 2024, proposing a completion-aware prediction framework, indicating that missing-data processing has become an important direction that cannot be ignored in the trajectory-prediction field.

### 2.2. Occluded-Trajectory Completion Methods

The goal of occluded-trajectory completion is to recover pedestrian positions in occluded or missing parts based on visible trajectories. According to methodological paradigms, existing research can be divided into three categories: traditional interpolation methods, deep generative completion methods, and prediction-oriented dedicated-completion methods.

Traditional Interpolation and Filtering Methods are computationally simple and require no additional training, and are capable of recovering missing trajectories to a certain extent. However, traditional methods rely on local continuity assumptions and lack deep modeling capabilities for understanding pedestrian-motion laws. When pedestrians accelerate, decelerate, or turn, simple interpolation tends to produce over-smoothed results that cannot accurately recover the nonlinear dynamic changes in real trajectories.

Deep Generative Completion Methods have made significant progress in recent years. BRITS [12] proposes a bidirectional recurrent imputation network that jointly models imputation and prediction and SAITS [13] utilizes self-attention mechanisms to learn dependencies between observed and missing values. With the development of diffusion models and Mamba structures, time-series completion has entered a new stage. CSDI [33] was the first to introduce conditional-score-diffusion models into time-series imputation, significantly improving probabilistic-imputation performance by explicitly modeling the distribution of missing values conditioned on observed values. SSD-TS [34] was the first to combine Mamba with diffusion models for time-series completion, proposing Bidirectional Attention Mamba (BAM) blocks and Channel Mamba Blocks (CMB), and achieving effective modeling of intra-sequence and inter-channel dependencies while maintaining linear complexity. MAC2STI [35] proposes a clustering-aware S6 structure specifically designed for missing patterns in traffic spatio-temporal data. These methods prove that the fusion of state-space models and generative models can effectively improve data-recovery capabilities under complex missing patterns.

Prediction-Oriented Dedicated Completion Methods differ from general time-series completion in that they need to simultaneously satisfy kinematic rationality and prediction friendliness. Qi et al. [36] proposed imitative non-autoregressive modeling to jointly optimize trajectory prediction and missing-value completion. The MS-TIP proposed by Chib et al. [32] is the first work to systematically study completion problems in the pedestrian-trajectory-prediction field, making prediction models robust to missing values in input trajectories through masking strategies and imputation-aware training. However, traditional interpolation methods struggle to characterize nonlinear motion changes while deep generative methods lack explicit modeling of pedestrian kinematic laws and rarely utilize physical quantities such as acceleration to guide the recovery process. Therefore, how to ensure the physical rationality of recovered trajectories at the motion level remains to be further explored.

### 2.3. Temporal Modeling Methods

Pedestrian trajectory prediction is essentially a time-series-modeling task, and effectively extracting motion patterns at different time scales is crucial for improving prediction performance. In recent years, temporal modeling methods have evolved from recurrent networks to convolutional networks, and then to Transformers and State-Space Models (SSM).

Recurrent Neural Networks and Convolutional Networks (LSTM and its variants) can process trajectory data in chronological order and learn dynamic change patterns, but their recursive structures limit long-sequence modeling efficiency while suffering from gradient-propagation difficulties. Temporal Convolutional Networks (TCN) expand temporal receptive fields through dilated convolutions, improving local motion-pattern-extraction capabilities. However, single-scale convolutions usually struggle to simultaneously capture short-term motion changes and long-term motion trends, making multi-scale temporal convolution a research hotspot.

Transformer and its variants utilize self-attention mechanisms to achieve global dependency modeling, succeeding in multiple sequence-prediction tasks. However, their computational complexity grows quadratically with sequence length, and their explicit modeling capability for local temporal patterns is limited. In recent years, for trajectory-prediction tasks, researchers have begun to explore lightweight attention mechanisms and local–global hybrid architectures to balance computational efficiency and modeling capability.

State-Space Models and Mamba Structures provide new solutions for long-term temporal modeling. The Mamba proposed by Gu and Dao [37] enhances long-sequence modeling capability through selective state-space mechanisms while maintaining linear computational complexity. To address the limitation of Mamba’s unidirectional scanning, bidirectional and multi-scale extensions have become important research directions: Bi-Mamba+ [38] achieves bidirectional modeling through forward-backward concatenation and gating mechanisms; ms-Mamba [39] proposes multi-scale parallel scanning to capture patterns at different time granularities; and Chimera [40] further proposes a two-dimensional time-channel scanning mechanism, establishing selective state spaces simultaneously in temporal and channel dimensions.

Hybrid architectures attempt to combine the advantages of Mamba with those of Transformer and convolutional architectures. MambaMixer [41] combines Mamba with MLP-Mixer to achieve joint modeling at the token and channel levels; ST-MambaSync [42], for spatiotemporal prediction tasks, synchronizes bidirectional Mamba with a spatiotemporal Transformer, thereby improving traffic-flow-prediction performance. In the trajectory-prediction field, Trajectory Mamba [8] introduces selective state-space mechanisms into trajectory prediction, verifying Mamba’s advantage in long-range temporal modeling with low computational overhead; TrajMamba [10] further adopts an ego-motion perspective, utilizing Mamba for egocentric trajectory modeling; and MMFlow [43] combines Mambaformer with single-step flow matching to achieve efficient multimodal trajectory generation. The W2W method proposed by Yang et al. [11] combines language models with reinforcement learning, providing a new state-space-modeling approach for trajectory prediction from the perspective of high-level semantic understanding.

Although the above Mamba methods perform excellently in general sequence tasks, for occlusion-recovered trajectory sequences, combining explicit motion-state information, multi-scale motion patterns, and bidirectional temporal dependency relationships, it still lacks in-depth research. Therefore, this paper further designs the Temporal Bi-Mamba module, enhancing the trajectory-prediction model’s understanding capability of historical-motion-state changes and overall trends under occlusion scenarios through forward and backward dual-temporal-direction modeling.

## 3. Methods

### 3.1. Problem Definition

A pedestrian-trajectory sequence within a historical time period

(1) $$X=\{x_{1},x_{2},\ldots ,x_{T}\}$$

where *T* represents the number of historical-observation frames, and $x_{t}$ represents the pedestrian trajectory state at frame *t*. In this paper, the trajectory state is represented as follows:

(2) $$x_{t}=\left[b_{t}^{x},\;b_{t}^{y},\;b_{t}^{w},\;b_{t}^{h}\right]$$

where $b_{t}^{x}$ and $b_{t}^{y}$ represent the center-point coordinates or position coordinates of the pedestrian bounding box, and $b_{t}^{w}$ and $b_{t}^{h}$ represent the width and height of the bounding box.

Under occlusion conditions, some historical frames are invisible. Define the occlusion mask as follows:

(3) $$M=\{m_{1},m_{2},\ldots ,m_{T}\}$$

where $m_{t}=1$ indicates visible and $m_{t}=0$ indicates occluded.

The occluded input trajectory can be represented as

(4) $$X_{\mathrm{occ}}=M⊙X$$

where ⊙ denotes element-wise multiplication. This paper adopts whole-frame occlusion, meaning when frame *t* is occluded, all dimensions of that frame’s trajectory are considered invisible. The goal of this paper is to first recover the complete historical trajectory $\hat{X}$ from the occluded trajectory $X_{\mathrm{occ}}$, and then predict the future trajectory $\hat{Y}$ based on the completed trajectory.

(5) $$\hat{X}=G\left(X_{\mathrm{occ}},M\right),\;\hat{Y}=F\left(\hat{X}\right)$$

### 3.2. Overall Framework

The proposed model consists of a Physics-Aware Residual Completion De-Occlusion Module and a Mamba Future-Trajectory-Prediction Module. First, the raw trajectory $X_{\mathrm{occ}}$ with missing frames is input into the physics-aware residual completion de-occlusion module to complete the occluded historical trajectory and recover the trajectory information of occluded frames; then, the recovered trajectory $\hat{X}$ is input into the improved Mamba future-trajectory-prediction model to obtain the future-trajectory-prediction result $\hat{Y}$.

**Figure 1:**
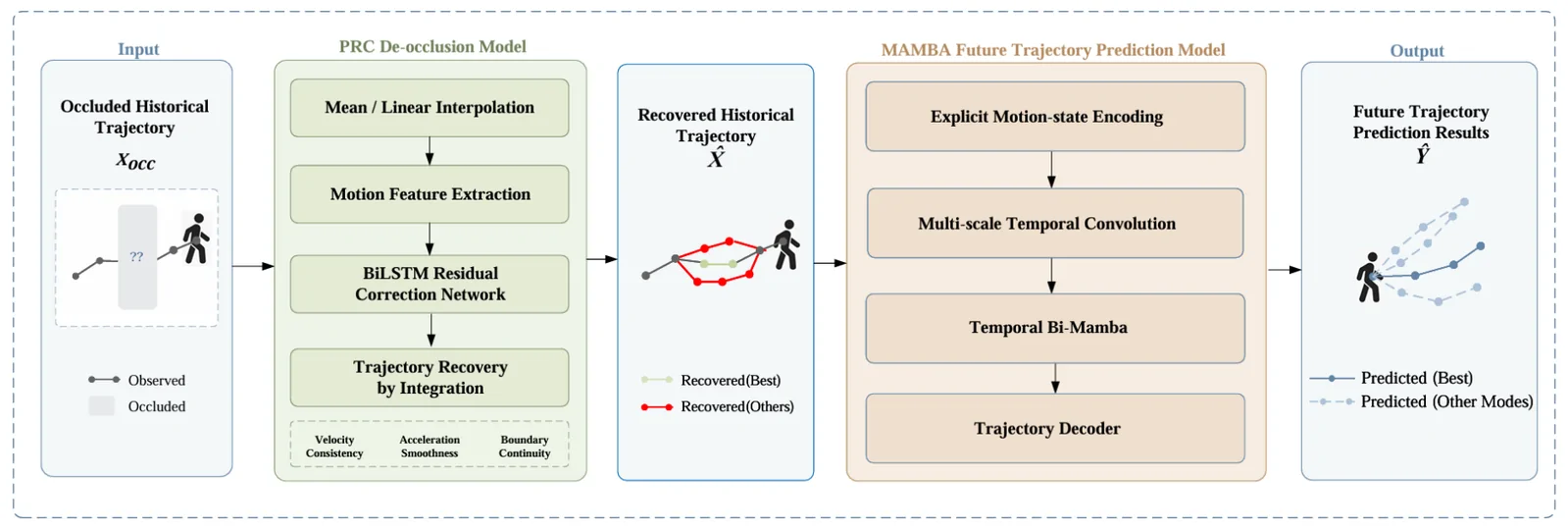
Overall framework of the proposed model.

The overall process is shown in Figure 1.

### 3.3. Physics-Aware Residual Completion De-Occlusion Module (PRCDM)

**Figure 2:**
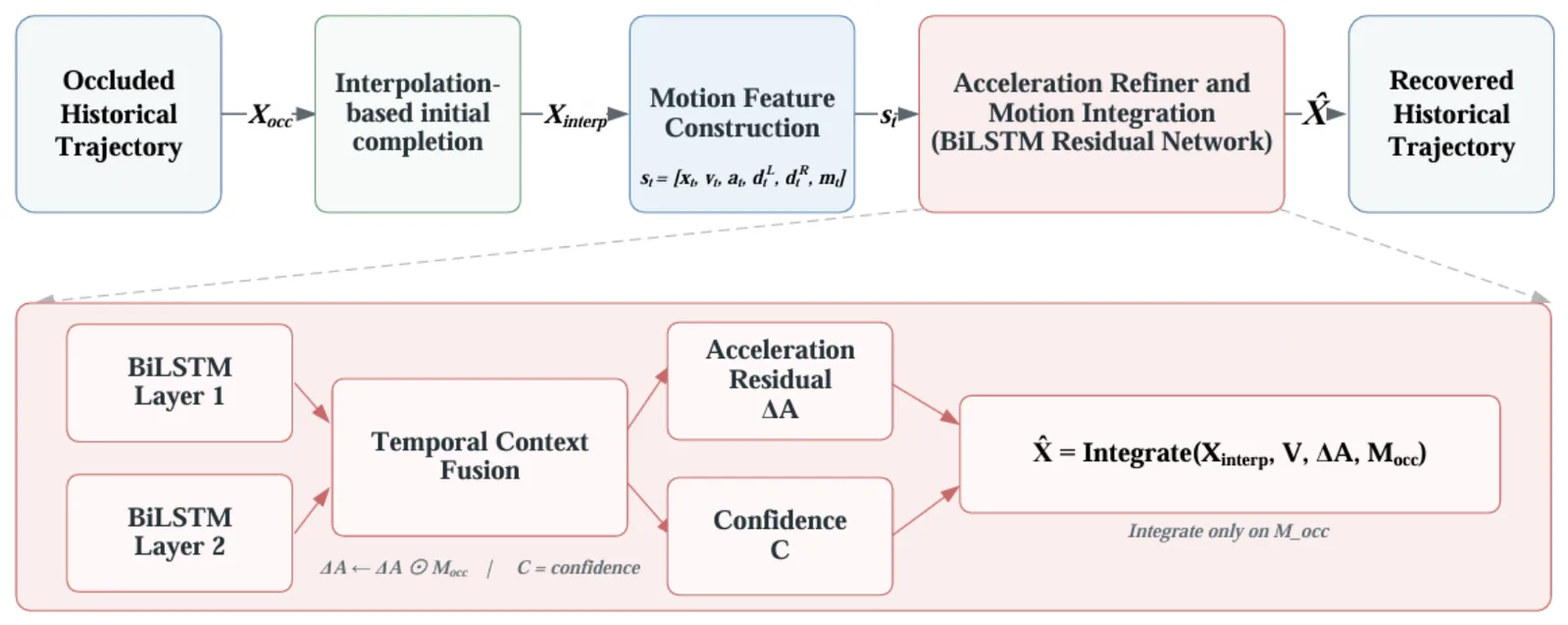
Structure of the proposed physics-aware residual completion de-occlusion module (PRCDM).

The overall pipeline of the proposed physics-aware residual completion de-occlusion model (PRCDM) is illustrated in Figure 2. The model follows the design philosophy of stable initialization first, then physics-aware correction: the mean interpolation method is simple, stable, and training-free, which provides a reasonable initial estimate for the occluded regions, but is difficult to recover the nonlinear motion changes that a pedestrian undergoes during occlusion. To this end, PRCDM adopts a two-stage cascaded structure. First, the interpolation initial completion (IIC) module constructs a smooth initial trajectory from the nearest visible frames before and after the occluded segment, providing a stable baseline for the subsequent correction. Then, based on the initial trajectory, motion features including position, velocity, acceleration, distance to the occlusion boundaries, and the occlusion mask are explicitly constructed, and the residual-correction network predicts the acceleration correction and its confidence for the occluded region, from which the completed full trajectory is recovered via motion integration. The entire model takes the occluded trajectory $X_{\mathrm{occ}}$ and the occlusion mask *M* as inputs and outputs the complete historical trajectory $\hat{X}$; residual correction is performed in the acceleration domain and recovered by integration, ensuring that the completed trajectory remains continuous in both position and velocity. The aforementioned components will be elaborated on in Section 3.3.1, Section 3.3.2, and Section 3.3.3, respectively, and correspond one-to-one to the processing flow in Figure 2.

#### 3.3.1. Interpolation-Based Initial Completion

In the de-occlusion stage, this paper first employs mean interpolation for preliminary recovery of missing trajectories (IIC). For occluded frames, initial estimates are calculated using coordinate information from visible trajectory frames before and after. Let frame *t* be an occluded frame, with its nearest visible frames before and after being $x_{t-1}$ and $x_{t+l}$, respectively. A basic interpolation form can be expressed as follows:

(6) $$x_{t}^{\mathrm{interp}}=\frac{x_{t-1}+x_{t+l}}{2}$$

When multiple consecutive frames are occluded, this paper constructs local interpolation trajectories based on visible boundaries before and after the occlusion segment, maintaining temporal continuity between the occluded segment and surrounding visible trajectories. Interpolation methods have the advantages of simplicity, stability, and no training requirement, providing a basis for subsequent residual correction.

However, interpolation results are essentially smooth estimates that struggle to accurately recover pedestrians’ nonlinear motion changes. Therefore, this paper further introduces a physics-aware residual completion model to perform motion-change-level correction based on interpolation results.

#### 3.3.2. Motion Feature Construction

To enable the completion model to not only focus on position coordinates but also understand the motion states of occluded trajectories, this paper constructs motion features based on interpolated trajectories. Features include position, velocity, acceleration, distances from the occluded frame to left and right visible boundaries, and the occlusion mask.

(7) $$v_{t}=x_{t}-x_{t-1},\;a_{t}=v_{t}-v_{t-1}$$

(8) $$s_{t}=\left[x_{t},\;v_{t},\;a_{t},\;d_{t}^{L},\;d_{t}^{R},\;m_{t}\right]$$

where $d_{t}^{L}$ and $d_{t}^{R}$ represent the distances from the current frame to the left and right visible boundaries, respectively, and $m_{t}$ represents the occlusion mask. By explicitly introducing these features, the model can determine the position of the occluded region within the entire trajectory and combine motion changes from visible trajectories to correct the occluded portion.

#### 3.3.3. Acceleration Residual Correction and Motion Integration (BiLSTM Residual Network)

The core of the proposed de-occlusion model is the residual-correction network. This module receives motion-feature sequences and outputs acceleration-correction amounts ΔA and confidence C for the occluded region. Unlike directly predicting coordinate residuals, acceleration correction adjusts the occluded segment from a motion-change perspective, making the completed trajectory more consistent with continuous motion laws.

(9) ΔA,C=Refiner(S,M)

To avoid unnecessary perturbation to visible trajectories, the acceleration correction amount is only applied to occluded regions in the following code:

(10) $$\Delta A=\Delta A⊙M_{\mathrm{occ}}$$

where $M_{\mathrm{occ}}$ represents the occluded region mask. Subsequently, based on the velocity V calculated from the interpolated trajectory and the predicted acceleration correction amount ΔA, the final trajectory is recovered through integration:

(11) $$\hat{X}=\mathrm{Integrate}\left(X_{\mathrm{interp}},V,\Delta A,M_{\mathrm{occ}}\right)$$

The advantages of this design are: interpolation results provide stable initial values, so the residual model only needs to learn motion changes that interpolation fails to express; meanwhile, the integration recovery approach makes the completed trajectory more continuous in both velocity and position.

### 3.4. MAMBA Future-Trajectory-Prediction Model

**Figure 3:**
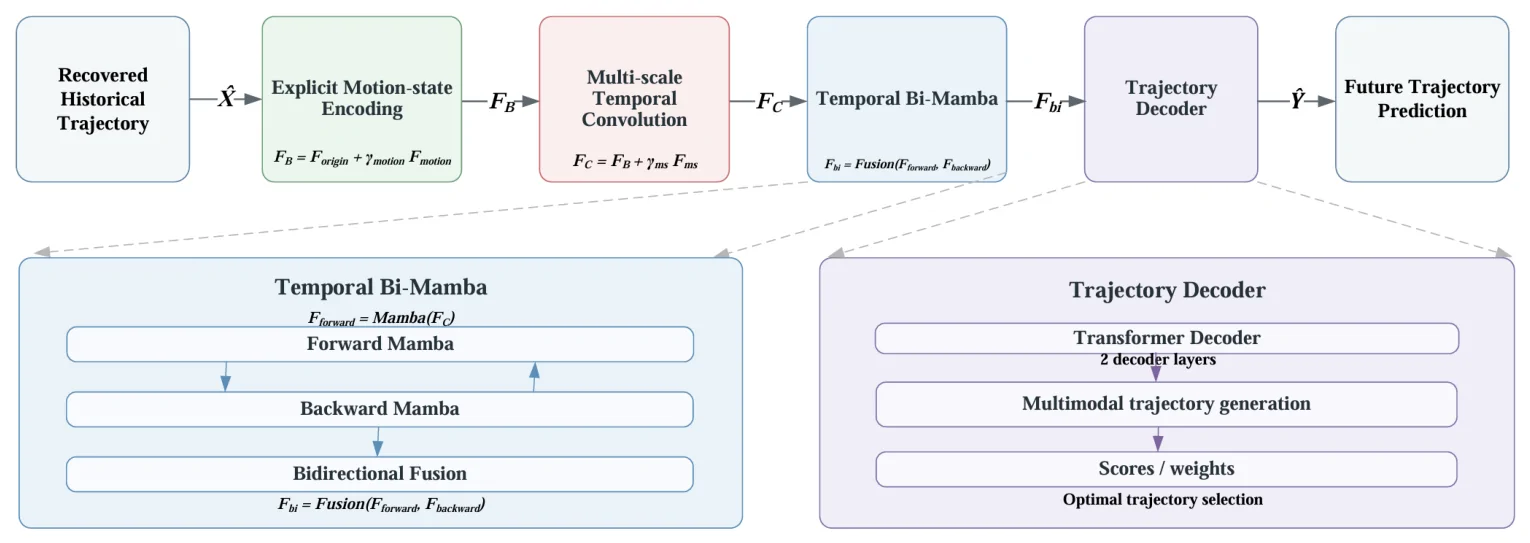
MAMBA future-trajectory-prediction model.

The architecture of the proposed future-trajectory-prediction model is shown in Figure 3.

#### 3.4.1. Explicit Motion-State Encoding (EME)

The explicit motion-state encoding module (EME) is used to explicitly encode pedestrian-motion states. Given a trajectory sequence $X=\{x_{1},x_{2},\ldots ,x_{T}\}$, velocity and acceleration are defined as

(12) $$v_{t}=x_{t}-x_{t-1},\;a_{t}=v_{t}-v_{t-1}$$

Thus, the motion-state feature at frame *t* can be represented as

(13) $$s_{t}=\left[x_{t},\;v_{t},\;a_{t}\right]$$

The motion-state features are mapped to high-dimensional space through an embedding layer to obtain $F_{\mathrm{motion}}$. The original DataEmbedding output is denoted as $F_{\mathrm{origin}}$. This paper adopts a weak residual-fusion approach to inject motion-state features into the original trajectory representation:

(14) $$F_{B}=F_{\mathrm{origin}}+\gamma_{\mathrm{motion}}\;F_{\mathrm{motion}}$$

This module mainly addresses the insufficient motion-state expression in baseline models, enabling the model to more directly perceive pedestrian-motion direction, velocity, and velocity-change trends.

#### 3.4.2. Multi-Scale Temporal Convolution (MTC)

The multi-scale temporal convolution module (MTC) takes $F_{B}$ as input, first performs layer normalization, and then uses multiple temporal convolution branches with different dilation rates to extract features along the temporal dimension. In the code, the dilation rates for multi-scale convolution branches are set to d=1,2,4.

(15) $$F_{1}={\mathrm{Conv}}_{d=1}\left(F_{B}\right),\;F_{2}={\mathrm{Conv}}_{d=2}\left(F_{B}\right),\;F_{4}={\mathrm{Conv}}_{d=4}\left(F_{B}\right)$$

where d=1 is used to capture short-term changes between adjacent time steps, d=2 is used to capture medium-range motion patterns, and d=4 is used to expand the temporal receptive field and capture longer-range local motion trends.

The fusion approach actually adopted in this paper is concatenation followed by linear mapping, i.e., concat + MLP/linear fusion. Outputs from different scale branches are first concatenated along the feature dimension, then remapped to the original feature dimension through a fusion network composed of linear layers, GELU activation, Dropout, and linear layers:

(16) $$F_{\mathrm{cat}}=\mathrm{Concat}\left(F_{1},F_{2},F_{4}\right)$$

(17) $$F_{\mathrm{ms}}=\mathrm{MLP}\left(F_{\mathrm{cat}}\right)$$

To avoid excessive perturbation to original features by the newly added module during initial training, this paper further adopts a weak residual fusion approach:

(18) $$F_{C}=F_{B}+\gamma_{\mathrm{ms}}\;F_{\mathrm{ms}}$$

In the code, $\gamma_{\mathrm{ms}}$ is initialized to $1\times 10^{-3}$ by default and remains unchanged during training. The multi-scale temporal convolution module (MTC) mainly addresses the insufficient extraction of local multi-scale temporal patterns in experiments, enabling the model to simultaneously capture short-term, medium-term, and longer-term motion changes.

#### 3.4.3. Temporal Bi-Mamba Module

The Temporal Bi-Mamba module (TMB) is used to enhance long-term temporal dependency modeling capability for trajectory sequences. Given the multi-scale temporal convolution output feature $F_{C}$, Temporal Bi-Mamba first models the trajectory sequence along the forward temporal direction:

(19) $$F_{\mathrm{forward}}=\mathrm{Mamba}\left(F_{C}\right)$$

Then, the sequence is reversed along the temporal dimension and modeled through another Mamba module in the backward direction:

(20) $$F_{\mathrm{backward}}=\mathrm{Reverse}\left(\mathrm{Mamba}\left(\mathrm{Reverse}(F_{C})\right)\right)$$

Finally, the forward and backward features are averaged or fused to obtain the bidirectional temporal representation:

(21) $$F_{\mathrm{bi}}=\mathrm{Fusion}\left(F_{\mathrm{forward}},F_{\mathrm{backward}}\right)$$

The introduction of Temporal Bi-Mamba enables analysis of trajectory sequences from both forward and backward temporal directions, enhancing the model’s understanding capability of historical-motion-state changes and overall motion trends.

The hyperparameters of the Temporal Bi-Mamba module are set as follows: state dimension $d_{\mathrm{state}}=16$, expansion factor $d_{\mathrm{expand}}=2$, convolution kernel width $d_{\mathrm{conv}}=4$, number of module layers N=2, and time-step rank $d_{t_{\mathrm{rank}}}=8$; RMSNorm is adopted for normalization and SiLU as the activation function. All model architectures and training hyperparameters in this paper are summarized in Table 1.

#### 3.4.4. Trajectory Decoder

The trajectory decoder is used to map high-dimensional bidirectional temporal features to specific future-trajectory coordinates. Specifically, to convert the bidirectional temporal representation $F_{\mathrm{bi}}$ output by Temporal Bi-Mamba into pedestrian positions for future $T_{f}$ frames, the decoder first aggregates $F_{\mathrm{bi}}$ along the temporal dimension to build a compact historical-motion context vector $h_{\mathrm{ctx}}$. Considering that pedestrian future motion has strong correlation with recent historical states, this paper adopts a combination of temporal-dimension average pooling and last-frame feature concatenation:

(22) $$h_{\mathrm{ctx}}=\left[\mathrm{MeanPool}\left(F_{\mathrm{bi}}\right);\;F_{\mathrm{bi}}\left[:,-1,:\right]\right]$$

where [·;·] denotes feature concatenation, MeanPool(·) takes the average along the temporal dimension to capture overall motion trends, and $F_{\mathrm{bi}}\left[:,-1,:\right]$ takes the bidirectional feature of the last frame of the historical sequence to enhance sensitivity to the current motion state.

Subsequently, $h_{\mathrm{ctx}}$ passes through a prediction head composed of two linear mappings, GELU activation, and Dropout, regressing displacement offsets for the future trajectory:

(23) $$\Delta \hat{Y}={\mathrm{MLP}}_{\mathrm{pred}}\left(h_{\mathrm{ctx}}\right)\in R^{T_{f}\times 4}$$

To further improve prediction stability and reduce regression difficulty, this paper adopts a residual decoding strategy. The decoder actually predicts displacement changes relative to the historical last frame $x_{T}$, with the final trajectory obtained by accumulating the historical last frame coordinates:

(24) $$\hat{Y}=x_{T}⊕\Delta \hat{Y}$$

where ⊕ denotes accumulating the historical last frame coordinate $x_{T}$ to the predicted offset of each future frame. This design enables the model to only learn incremental changes relative to the current state, avoiding numerical instability from directly regressing absolute coordinates during early training, while ensuring spatio-temporal continuity of predicted trajectories.

Echoing the weak residual fusion strategy adopted in the EME and MTC modules, the trajectory decoder also follows the design principle of lightweight, stable, and low-perturbation, without introducing complex autoregressive generation or multimodal sampling mechanisms. Instead, it directly outputs future trajectories $\hat{Y}=\left\{{\hat{y}}_{1},{\hat{y}}_{2},\ldots ,{\hat{y}}_{T_{f}}\right\}$ through deterministic single-step decoding. This concise decoding approach ensures prediction accuracy while significantly reducing model inference latency, making it more suitable for real-time deployment in autonomous-driving scenarios.

### 3.5. Loss-Function Design

The overall model consists of an occluded-trajectory completion module and a future-trajectory-prediction module; therefore, the training objective needs to simultaneously consider historical-trajectory-recovery accuracy and future-trajectory-prediction performance. To enable the model to effectively recover motion information under occlusion conditions and further improve future-trajectory-prediction accuracy, this paper designs a joint optimization-loss function.

#### 3.5.1. Completion-Loss Function

The total loss of the completion module consists of position-reconstruction loss, velocity-consistency loss, confidence-supervision loss, and confidence-sparsity constraint.

The position-reconstruction loss constrains the coordinate error between completed trajectories and real trajectories in occluded regions:

(25) $$L_{\mathrm{pos}}=\mathrm{MSE}\left(\hat{X}⊙M_{\mathrm{occ}},\;X⊙M_{\mathrm{occ}}\right)$$

The velocity-consistency loss constrains motion continuity of completed trajectories along the temporal dimension:

(26) $$L_{\mathrm{vel}}=\mathrm{MSE}\left(\hat{V}⊙M_{\mathrm{occ}},\;V⊙M_{\mathrm{occ}}\right)$$

The confidence-supervision loss constrains the model-output confidence C, enabling it to reflect the reliability of completion results. Target confidence is constructed based on the frame-wise error $e_{t}$ between completed trajectories and real trajectories:

(27) $$e_{t}=\mathrm{mean}\left(|x_{t}-{\hat{x}}_{t}|\right),\;C_{t}^{\mathrm{tgt}}=\exp\left(-100\;e_{t}\right)$$

(28) $$L_{\mathrm{conf}}=\mathrm{MSE}\left(C,C^{\mathrm{tgt}}\right)$$

The confidence-sparsity constraint controls the confidence distribution in non-occluded regions, making the model focus more on truly occluded regions:

(29) $$L_{\mathrm{sparse}}=\frac{\sum \left(\left(1-C\right)⊙\left(1-M_{\mathrm{occ}}\right)\right)}{\sum (1-M_{\mathrm{occ}})+\varepsilon }$$

The final total completion loss is

(30) $$L_{\mathrm{imp}}=\lambda_{\mathrm{pos}}L_{\mathrm{pos}}+\lambda_{\mathrm{vel}}L_{\mathrm{vel}}+\lambda_{\mathrm{conf}}L_{\mathrm{conf}}+\lambda_{\mathrm{sparse}}L_{\mathrm{sparse}}$$

In the code, default weights are set to $\lambda_{\mathrm{pos}}=1.0$, $\lambda_{\mathrm{vel}}=0.5$, $\lambda_{\mathrm{conf}}=0.1$, $\lambda_{\mathrm{sparse}}=0.01$, therefore, the actual training objective is

(31) $$L_{\mathrm{imp}}=L_{\mathrm{pos}}+0.5\;L_{\mathrm{vel}}+0.1\;L_{\mathrm{conf}}+0.01\;L_{\mathrm{sparse}}$$

During training, the model generates occluded samples using a curriculum-learning approach, gradually increasing occlusion ratios from low to high to improve the completion model’s robustness under different occlusion intensities.

#### 3.5.2. Future-Trajectory-Prediction Loss

After completing historical-trajectory recovery, this paper inputs the completed trajectory into the MAMBA future-trajectory-prediction network to generate future-trajectory-prediction results.

Let the real future trajectory be

(32) $$Y=\{y_{1},y_{2},\ldots ,y_{T}\}$$

The predicted future trajectory is

(33) $$\hat{Y}=\left\{{\hat{y}}_{1},{\hat{y}}_{2},\ldots ,{\hat{y}}_{T}\right\}$$

This paper adopts average displacement error as the future-trajectory-prediction-optimization objective:

(34) $$L_{\mathrm{pred}}=\frac{1}{T}\sum_{t}{∥y_{t}-{\hat{y}}_{t}∥}_{2}$$

This loss can directly constrain the position deviation between predicted trajectories and real trajectories, enabling the model to learn future-motion trends.

#### 3.5.3. Joint Optimization Objective

To simultaneously optimize trajectory-recovery capability and future-prediction performance, this paper adopts a multi-task joint-training strategy. The total loss function is defined as follows:

(35) $$L_{\mathrm{total}}=L_{\mathrm{imp}}+\lambda_{\mathrm{pred}}L_{\mathrm{pred}}$$

where $L_{\mathrm{imp}}$ represents the occluded-trajectory completion loss, $L_{\mathrm{pred}}$ represents the future-trajectory-prediction loss, and $\lambda_{\mathrm{pred}}$ is used to balance the optimization ratio between the two tasks.

Through joint optimization, the model can simultaneously learn occlusion-region motion-recovery capability and future-trajectory-prediction capability during training, enabling recovered historical trajectories to more effectively serve subsequent prediction tasks, thereby improving overall pedestrian-future-trajectory-prediction performance under occlusion conditions.

## 4. Experiments and Results

### 4.1. Experimental Settings

#### 4.1.1. Datasets

To verify the effectiveness of the proposed method, experiments were conducted on the publicly available Joint Attention in Autonomous Driving (JAAD) [44] and Pedestrian Intention Estimation (PIE) [45] datasets. Both datasets comprise data collected from onboard forward-facing cameras, reflecting dynamic interactions between pedestrians and the ego vehicle in autonomous-driving scenarios. The JAAD dataset contains 2800 pedestrian trajectories captured by onboard forward-facing cameras, annotated at 30 Hz. Similarly, the PIE dataset contains 1835 pedestrian trajectories, also annotated at 30 Hz. Both datasets provide video frames, pedestrian bounding-box coordinates, and ego vehicle-motion information. To ensure a fair comparison with existing methods, we follow the official standard splits for the JAAD and PIE datasets, which is consistent with prior work: the training, validation, and test sets are split at ratios of 50%, 10%, and 40%, respectively. Specifically, the input historical-sequence length is $T_{h}=15$ (0.5 s), and the pedestrian trajectory-prediction ranges are $T_{f}=\left\{15,30,45\right\}$ (0.5 s, 1.0 s, and 1.5 s).

This paper sets the historical-observation length to $T_{h}=15$ (0.5 s), based mainly on the following three considerations. First, to ensure fair comparison, this paper strictly follows the data-splitting protocol commonly used by existing pedestrian trajectory-prediction methods on the JAAD and PIE datasets, and compares it with all baseline methods under the same input observation length ($T_{h}=15$) and prediction horizon ($T_{f}=15,30,45$), avoiding unfair comparison results caused by different input lengths. Second, at a 30 Hz sampling rate, the 0.5 s historical window contains 15 trajectory-state frames, which is sufficient to characterize the short-term motion dynamics of pedestrians and to provide adequate motion context for future prediction over 0.5–1.5 s. Third, this paper focuses on occlusion scenarios. Under occlusion, the information effectiveness of historical observations depends on the number of visible frames rather than the total number of frames; a longer historical sequence may contain more occluded frames, increasing the difficulty of completion. In addition, the proposed multi-scale temporal convolution and Temporal Bi-Mamba modules are naturally adaptive to different input lengths, so the model’s performance will not fluctuate significantly with the input length.

#### 4.1.2. Occlusion Settings

To verify the model’s robustness under occlusion scenarios, this paper artificially constructs occlusion in historical trajectories. The occlusion method adopts whole-frame occlusion, meaning that when a historical frame is selected as an occluded frame, all dimensions of that frame’s trajectory are occluded. This paper sets multiple occlusion ratios for experiments: r∈{5%,10%,15%,20%,30%,40%}.

The occlusion ratio represents the proportion of occluded frames to total historical frames in the historical-observation trajectory. Each group of experiments is repeated three times, and the average results are taken as the final experimental results.

In this paper, two occlusion modes are configured: (1) *Random whole-frame occlusion*: a ratio *r* of frames (i.e., r·T frames) is independently and randomly selected for masking, simulating unstructured missing data such as detector misdetections; and (2) *Continuous segment occlusion*: a continuous segment of r·T frames is occluded at ratio *r*, with the starting position of the occluded segment being randomly chosen, simulating structural occlusion caused by vehicles, buildings, and so on. The two modes are evaluated in pairs under six occlusion ratios ranging from 5% to 40%. It should be noted that at the 5% ratio, continuous occlusion degenerates into single-frame occlusion and, thus, becomes equivalent to random occlusion.

#### 4.1.3. Evaluation Metrics

This paper adopts ADE (0.5 s), ADE (1.0 s), ADE (1.5 s), FDE, C-ADE (0.5 s), C-ADE (1.0 s), C-ADE (1.5 s), and C-FDE as the main evaluation metrics.

ADE represents Average Displacement Error, measuring the average distance error between predicted trajectories and real trajectories. For future time length $T_{f}$, ADE is defined as

(36) $$\mathrm{ADE}=\frac{1}{T_{f}}\sum_{t=1}^{T_{f}}{∥y_{t}-{\hat{y}}_{t}∥}_{2}$$

FDE represents Final Displacement Error, measuring the distance error between the predicted endpoint and the real endpoint:

(37) $$\mathrm{FDE}=∥y_{T_{f}}-{\hat{y}}_{T_{f}}{∥}_{2}$$

C represents center-point error, C-ADE denotes the average displacement error calculated based on pedestrian center-point coordinates, and C-FDE denotes the final displacement error calculated based on pedestrian center-point coordinates.

#### 4.1.4. Experimental Details

The model is trained on a single RTX 3060 GPU using the Adam optimizer, with the learning rate set to 0.0004 and decayed according to validation loss. The pedestrian trajectory dimension is $N_{b}=4$, the historical-trajectory length is 15 frames, the future prediction length is 45 frames, training lasts for 50 epochs, and the batch size is 128.

The complete configuration of all model architecture hyperparameters and training hyperparameters in this paper is shown in Table 1. Specifically, the Mamba-related hyperparameters are kept consistent with the original Mamba implementation, and the bidirectional scanning structure does not alter its internal parameter configuration. The loss function is $L_{\mathrm{total}}=L_{\mathrm{imp}}+\lambda_{\mathrm{pred}}\cdot L_{\mathrm{pred}}$, where the completion-loss weight is $\gamma_{\mathrm{ms}}=1\times 10^{-3}$, and the prediction-loss weight is $\lambda_{\mathrm{pred}}=1.0$.

During the training stage, apart from curriculum learning, no additional data augmentation strategies are applied to the trajectory data in this paper. The random generation of occlusion masks itself serves as an implicit form of data augmentation: during training, historical-trajectory frames are randomly masked to synthesize occluded training samples, and the occlusion ratio gradually increases from low to high under the curriculum learning strategy, covering both the random whole-frame occlusion and continuous segment occlusion modes, which effectively enriches the diversity of training samples and enhances the model’s robustness to different occlusion modes. To ensure a fair comparison, all baseline methods are trained and evaluated under the same data augmentation settings, and each set of experiments is repeated three times with the average results reported.

### 4.2. Quantitative Analysis

To verify the effectiveness of the proposed future-trajectory-prediction module under complete trajectory input conditions, this paper first compares the proposed method with representative pedestrian trajectory-prediction methods in recent years under occlusion-free conditions. Experimental results are shown in Table 2.

From Table 2, it can be seen that the proposed method achieves optimal performance on all evaluation metrics. On the JAAD dataset, the proposed method’s ADE 0.5 s, ADE 1.0 s, and ADE 1.5 s reach 25, 57, and 112, respectively, representing reductions of 10.7%, 9.5%, and 9.7% compared to the currently better-performing NIM-STGCN method. On the PIE dataset, the proposed method also achieves optimal performance. The ADE 0.5 s, ADE 1.0 s, and ADE 1.5 s of the proposed method reach 13, 28, and 52, respectively, representing reductions of 7.1%, 9.7%, and 8.8% compared to NIM-STGCN. Meanwhile, on the C-FDE metric, which focuses more on long-term prediction accuracy, the proposed method achieves 238 and 84 on the JAAD and PIE datasets, respectively, representing reductions of 13.5% and 13.4% compared to NIM-STGCN. The results indicate that the proposed MAMBA future-trajectory-prediction module can effectively model long-term temporal dependency relationships in pedestrian-motion processes and improve future-trajectory-prediction accuracy.

To further verify the robustness of the proposed method under different occlusion conditions, this paper compares it with existing occlusion-aware trajectory-prediction methods, including MS-TIP [32] and the “mean interpolation + SGNet” combined baseline. All methods are evaluated under the same protocol: the same data split, the same input length, six occlusion ratios ranging from 5% to 40% covering both random and consecutive occlusion modes, and each setting is repeated three times and averaged. The results are shown in Table 3 and Table 4.

RCN-MAMBA achieves the lowest errors across all six occlusion ratios (5–40%) under both occlusion modes. Taking the JAAD dataset at 40% occlusion as an example: under random occlusion, the ADE1.5s and FDE of RCN-MAMBA are 188.6 and 391.5, respectively, which are 27.8% and 29.9% lower than those of MS-TIP (261.3 and 558.7), and 36.8% and 38.7% lower than those of the “mean interpolation + SGNet” baseline (298.6 and 638.2); under continuous occlusion, the ADE1.5s and FDE of RCN-MAMBA are 227.9 and 476.3, which are 28.1% and 30.2% lower than those of MS-TIP (316.8 and 682.4). As an occlusion-aware method specifically designed for missing data, MS-TIP outperforms the “mean interpolation + SGNet” combined baseline but still lags behind the proposed method, indicating that a general strong predictor equipped with only simple interpolation can hardly cope with structural missingness. The joint design of physics-aware residual completion and Temporal Bi-Mamba bidirectional temporal modeling maintains optimal performance under both random and continuous occlusion. As the occlusion ratio increases and the occlusion mode shifts from random to continuous, the relative advantage of the proposed method further expands. The PIE dataset exhibits a completely consistent trend.

Comparing the two occlusion modes ratio by ratio, consecutive occlusion is more difficult than random occlusion at all ratios. Taking the JAAD dataset as an example, at 40% occlusion, the ADE1.5s of Baseline increases from 342.6 (random) to 424.5 (consecutive), and the FDE increases from 731.5 to 912.7, indicating that missing information concentrated in space (time) causes greater damage to the prediction model than uniformly distributed missing data. The error gap between the consecutive and random modes increases monotonically with the occlusion ratio, confirming that interpolation-based methods suffer from more severe over-smoothing in long occlusion segments. The relative error reduction for RCN-MAMBA over Baseline under consecutive occlusion (42–50%) is generally higher than that under random occlusion (40–47%), indicating that the physics-aware residual completion and the bidirectional temporal modeling of Temporal Bi-Mamba are more targeted at structural missing data. The PIE dataset exhibits the same trend.

The experimental results demonstrate that the proposed method can effectively alleviate the problem of missing historical-trajectory information caused by occlusion, and the model has strong adaptability to different degrees of occlusion.

To further validate the effectiveness of the proposed trajectory de-occlusion module and investigate the influence of different initial interpolation schemes on the completion and prediction performance, we compare three pure interpolation methods, namely mean interpolation, linear interpolation, and cubic spline interpolation, with the proposed physics-aware residual completion method (PRC) being initialized by mean interpolation and its spline-initialized variant. The experimental results are shown in Table 5 and Table 6. Specifically, Mean, Linear, and Spline denote the pure interpolation completion results; PRC denotes the proposed “mean interpolation initialization + residual-correction” method; and Spline+Residual is a control variant initialized by spline interpolation, which is used to verify the influence of the initial interpolation scheme on the complete pipeline.

It can be observed that, on both datasets and under all occlusion ratios, the PRC method consistently and significantly outperforms any pure interpolation method. Among the three pure interpolation methods, cubic spline interpolation achieves the lowest errors, followed by linear interpolation. In contrast, mean interpolation yields the largest errors. The gaps among them widen as the occlusion ratio increases: at 40% occlusion on the JAAD dataset, the completion MAE of Spline is 53.6, which is 13.1% lower than that of Mean (61.7), and the ADE decreases from 82.7 to 73.2, a reduction of 11.5%. This indicates that when the continuous occluded segment becomes longer, the practice of mean interpolation, fixing all frames within the occluded segment to the Mean of the two boundary endpoints, amplifies the over-smoothing error. In contrast, linear interpolation, which is weighted by temporal position, and spline interpolation, which exploits smoothness constraints, can partially alleviate this problem. However, even the best spline interpolation still performs significantly worse than the proposed PRC method: at 40% occlusion on the JAAD dataset, the ADE of PRC is 49.8, which is 32.0% lower than that of Spline (73.2), and the FDE decreases from 152.8 to 105.7 (a reduction of 30.8%); and at 40% occlusion on the PIE dataset, the ADE of PRC is 31.0% lower than that of Spline. As the occlusion ratio increases, the performance gap between the pure interpolation methods and the proposed PRC method widens, indicating that simple interpolation can only recover the trajectory’s overall trend. In contrast, PRC can learn the dynamic deviation between the ground-truth trajectory and the interpolated trajectory, yielding recovery results that more closely conform to real motion patterns.

The advantage of spline interpolation at the pure interpolation level is not preserved in the complete pipeline; instead, it is reversed. After incorporating residual correction, Spline+Residual consistently underperforms the proposed PRC method at all occlusion ratios, and the gap widens as the occlusion ratio increases. Taking 40% occlusion as an example, the ADE is relatively degraded by 6.8% on the JAAD dataset and 6.9% on the PIE dataset. This phenomenon is consistent with the mechanism of residual learning: the deviation left by mean interpolation is a low-frequency, approximately monotonic smooth signal that is easy for the residual network to fit; in contrast, the spurious curvature introduced by spline interpolation within the occluded segment strengthens as the segment lengthens, and the residual network must first cancel this spurious structure before learning the true motion, which increases the learning difficulty. This indicates that the advantage of complex interpolation methods exists only in the absence of a learning mechanism and instead becomes a burden within the framework of stable initialization, followed by physics-aware correction. The proposed design of mean interpolation initialization + residual correction outperforms, directly adopting more complex interpolation in both performance and simplicity, and validating the rationality of the choice of the initial interpolation scheme.

### 4.3. Ablation Study

To analyze the contribution of each module to the final prediction performance, this paper conducts ablation experiments under 40% occlusion conditions, progressively adding the de-occlusion module, motion-state encoding module (EME), multi-scale temporal convolution module (MTC), and Temporal Bi-Mamba module (TBM). Experimental results are shown in Table 7 and Table 8.

From Table 6, it can be seen that when only the Baseline model is used, prediction errors are largest due to substantial missing information in the input trajectory, with ADE 1.5s reaching 342.6 and C-FDE reaching 604.2. After adding Mean interpolation, model performance improves, with ADE 1.5s decreasing to 293.4, indicating that simple trajectory completion can alleviate some information missing problems; however, due to the mean interpolation’s inability to recover real motion changes, the performance improvement is limited. After further adding the residual-correction module, ADE 1.5s dramatically decreases from 293.4 to 214.7, and FDE decreases from 621.8 to 452.6, proving that the residual learning mechanism can effectively recover dynamic motion information in occluded regions. After adding the motion-state encoding module (EME) and multi-scale temporal convolution module (MTC), model performance further improves, with ADE 1.5s decreasing to 191.6 and 165.2, respectively, indicating that more effective temporal modeling capability can improve future prediction accuracy on recovered trajectories. Finally, after adding the Temporal Bi-Mamba module (TBM) to the complete model, ADE 1.5s further decreases to 132.4 and C-FDE decreases to 238.6, achieving the best results.

From Table 7, it can be observed that each module shows a consistent contribution trend on the PIE dataset as on JAAD. The Baseline model’s ADE 1.5s reaches 159.1 and C-FDE reaches 213.3 under 40% occlusion; after adding Mean interpolation, ADE 1.5s decreases to 136.2; and after introducing Residual correction, it further decreases to 99.7, a reduction of 26.8%. Subsequently adding EME, MTC, and TBM modules progressively, ADE 1.5s decreases to 89.0, 76.7, and 61.5, respectively, and C-FDE decreases to 118.1, 101.1, and 84.2, respectively.

The ablation study verifies the effectiveness of each component module in this paper, while demonstrating that there is a clear complementary relationship between the de-occlusion recovery and future-trajectory-prediction stages, and their combination can fully enhance prediction performance in occluded environments.

To quantify the statistical significance of the incremental contribution of each module, we performed per-trajectory pairwise *t*-tests between adjacent configurations in Table 7 and Table 8 for the same test trajectories, with the Holm–Bonferroni correction applied to control for multiple comparisons. The results are shown in Table 9 and Table 10.

It can be observed that, on both the JAAD and PIE datasets, the addition of the de-occlusion modules (+Mean interpolation and +Residual correction) and each prediction module (+EME, +MTC, and +TBM) yields statistically significant performance improvements across all five metrics (p<0.001). Among them, the contribution of residual correction is the most prominent (on the JAAD dataset, the ADE1.5s decreases by 78.7 on average and the C-FDE by 143.6), and the incremental contributions of EME, MTC, and TBM are also significant (the ADE1.5s decreases by 23.1, 26.4, and 32.8, respectively). Furthermore, the 95% confidence intervals corresponding to the incremental improvements of adjacent configurations all exclude 0, further ruling out the possibility that the performance gains arise from random fluctuations. These results demonstrate that the contributions of the constituent modules of the proposed method are statistically reliable and validate the consistent effectiveness of the joint two-stage design of completion and prediction under occlusion scenarios.

Regarding the choice of the occlusion ratio for the ablation study, we adopt 40%, the most severe occlusion setting within the evaluation range, for the following reasons. First, at 40% occlusion, the contributions of each module are the most significant and easiest to distinguish: if a module can still bring consistent performance improvements under the most severe occlusion condition, it will also be effective at lower occlusion ratios; whereas at low occlusion ratios, the number of missing frames is small, leaving limited room for improvement for the completion module, and the differences among modules diminish accordingly, making it difficult to reflect the true contribution of each module. Second, as shown in Table 3 and Table 4, the error reduction in the complete method relative to Baseline increases monotonically with the occlusion ratio, indicating that the contributions of each module strengthen as the occlusion difficulty increases, and the ablation results at 40% represent the most significant case of module contributions. Third, Table 3 and Table 4 also show that the proposed method significantly outperforms Baseline at lower occlusion ratios such as 10% and 20%, demonstrating that the joint two-stage design of completion and prediction remains effective in common occlusion scenarios. In summary, the ablation results at 40% occlusion can represent the contribution patterns of each module within the evaluation range of this paper.

To further analyze the contribution of each loss term in the joint completion loss, we conduct a loss-term ablation study on the JAAD dataset at 40% occlusion. The position reconstruction loss $L_{\mathrm{pos}}$, which is the core of the completion performance, is retained; while keeping the other loss terms unchanged, we remove the velocity consistency loss $L_{\mathrm{vel}}$, the confidence supervision loss $L_{\mathrm{conf}}$, and the confidence sparsity constraint $L_{\mathrm{sparse}}$ one by one. The experimental results are shown in Table 11.

From Table 11, after removing $L_{\mathrm{vel}}$, the ADE1.5s increases from 132.4 to 139.5, a relative increase of 5.4%, indicating that the velocity consistency loss effectively constrains the motion continuity of the completed trajectory along the temporal dimension; after removing $L_{\mathrm{conf}}$, the ADE1.5s increases from 132.4 to 136.2, a relative increase of 2.9%, indicating that the confidence supervision loss can guide the model to focus on the occluded regions that are difficult to recover; and after removing $L_{\mathrm{sparse}}$, the ADE1.5s increases from 132.4 to 133.2, a relative increase of 0.6%, indicating that the sparsity constraint, as an auxiliary regularization term, mainly encourages the confidence to concentrate on the genuinely occluded regions. Statistical tests show that the performance changes caused by removing $L_{\mathrm{vel}}$ and $L_{\mathrm{conf}}$ are statistically significant. In contrast, the difference from removing $L_{\mathrm{sparse}}$ is not statistically significant, indicating that it makes a limited contribution as an auxiliary regularization term without harming overall performance. These results validate the rationality of the hierarchical weighting scheme.

### 4.4. Qualitative Analysis

To more intuitively demonstrate the advantages of the proposed method under occlusion scenarios, this paper further analyzes the model’s recovery capability and prediction capability through trajectory-visualization experiments.

#### 4.4.1. Occluded-Trajectory Recovery Visualization

**Figure 4:**
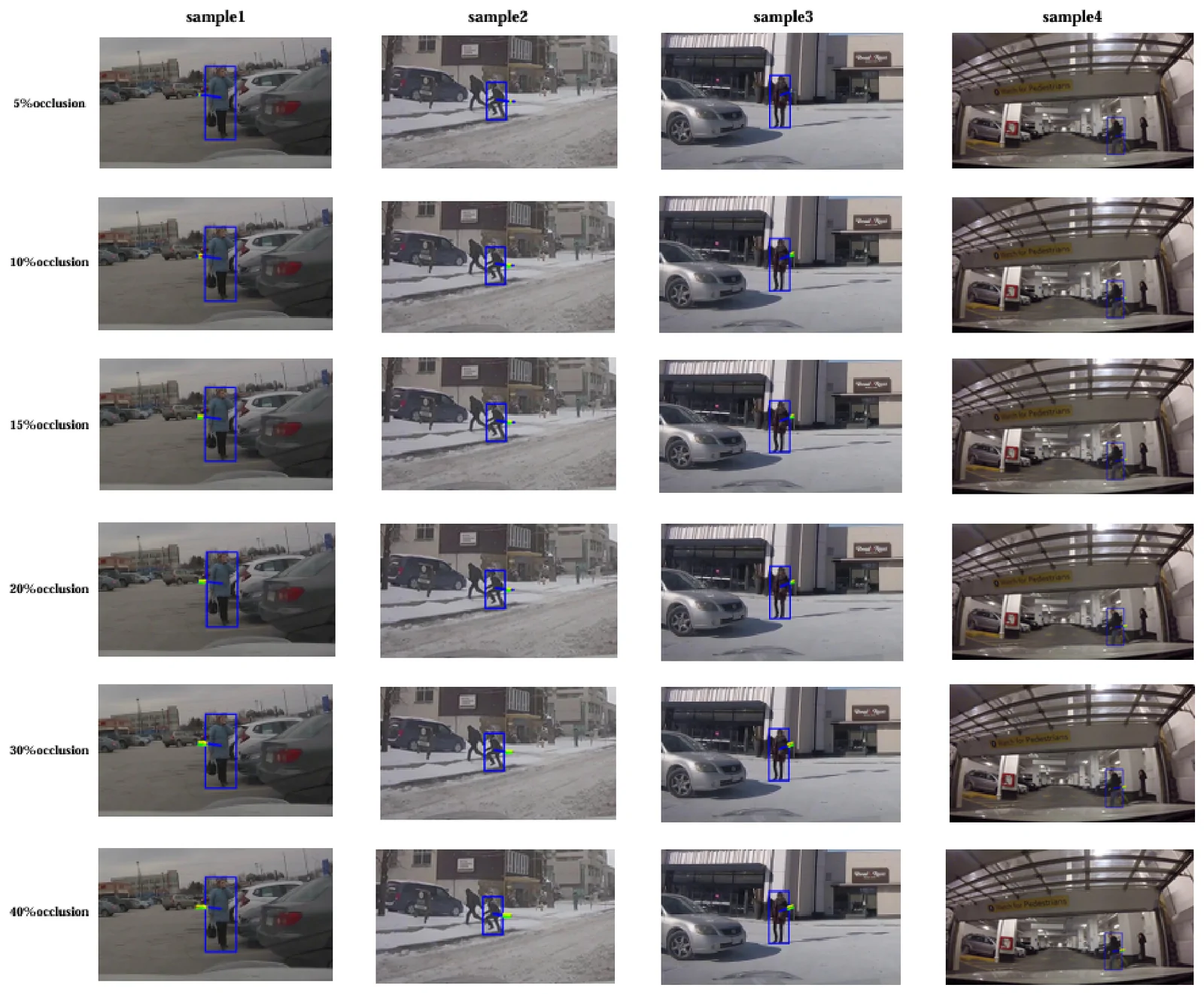
Recovery results of different methods for pedestrians’ historical trajectories under occlusion conditions. Blue solid line represents the real trajectory; red region represents the occluded trajectory; yellow curve represents the mean interpolation recovery result; and green curve represents the RCN recovery result of this paper.

As shown in Figure 4, the recovery results of different methods for pedestrians’ historical trajectories under occlusion conditions are presented.

As shown in Figure 4, the proposed method exhibits a clear advantage in terms of occluded-trajectory recovery. Taking the first example in Figure 4 as an illustration, the pedestrian is continuously occluded in the middle of the historical trajectory, and significant velocity changes and direction adjustments occur during the occlusion: the recovery curve of mean interpolation is approximately a flat straight segment within the occluded region, failing to reflect the motion changes during the occlusion and deviating significantly from the ground-truth trajectory. In contrast, the trajectory recovered by the proposed RCN method smoothly reproduces the pedestrian’s turning process within the occluded region and closely matches the ground-truth trajectory. The reason is that, based on the stable initial values provided by mean interpolation, the RCN method explicitly constructs motion features such as position, velocity, and acceleration, predicts the acceleration corrections for the occluded region through the residual-correction network, and recovers the trajectory via motion integration, thereby effectively compensating for the nonlinear motion changes that simple interpolation cannot recover. The above phenomenon is consistent with the quantitative conclusion in Table 5 and Table 6 that the proposed PRC method outperforms pure interpolation methods at all occlusion ratios, indicating that the residual-correction mechanism can learn the dynamic difference between the interpolation result and the ground-truth trajectory, yielding recovery results that better conform to the real motion patterns.

#### 4.4.2. Future-Trajectory-Prediction Visualization

**Figure 5:**
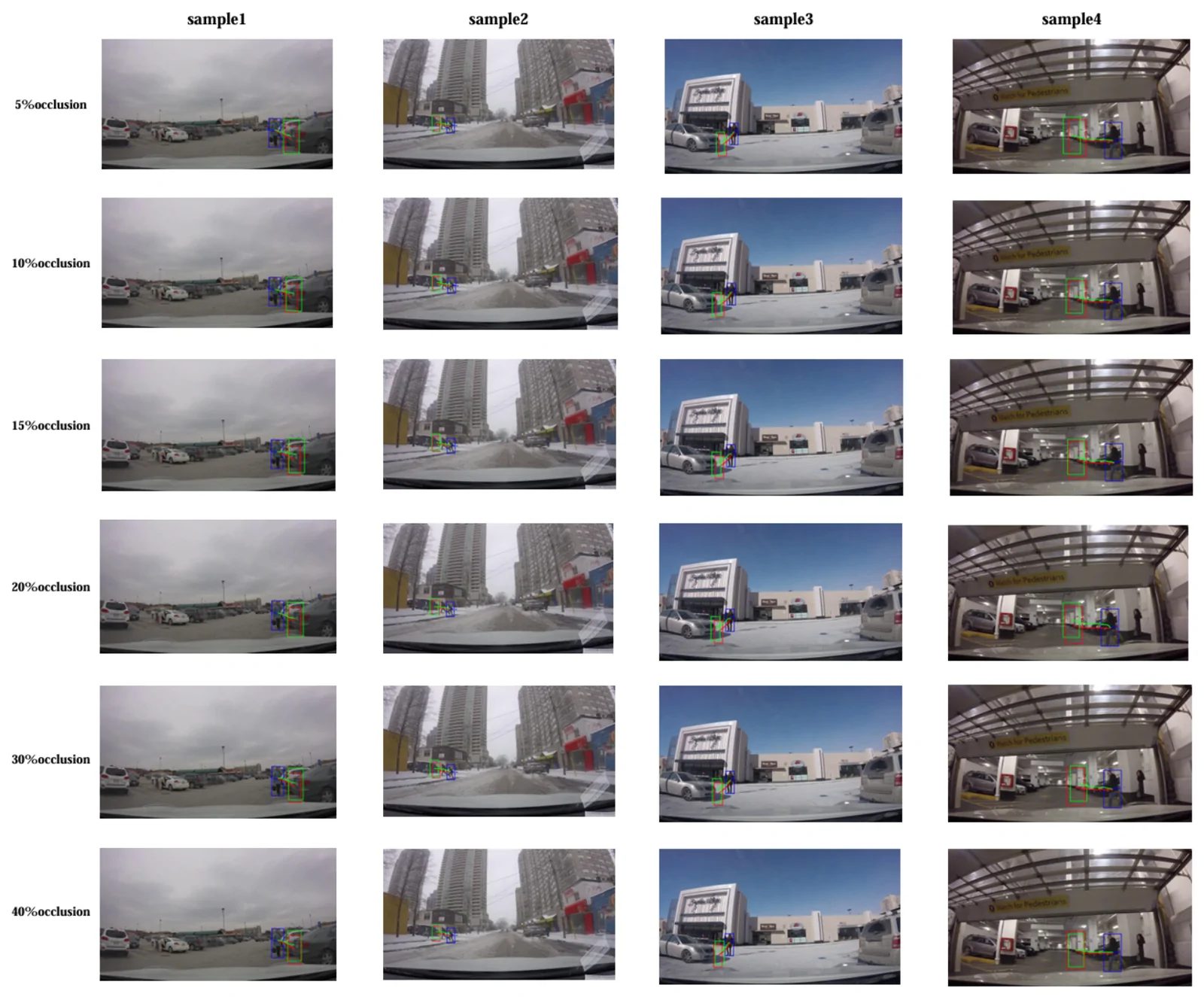
Future-trajectory-prediction results of RCN-MAMBA method under occlusion environments. Historical-observation trajectories are represented in blue; real future trajectories are represented in red; and model predicted trajectories are represented in green.

As shown in Figure 5, the future-trajectory-prediction results of the RCN-MAMBA method under occlusion environments are presented.

From Figure 5, the advantage of the proposed method for future-trajectory prediction under occlusion is evident. The proposed RCN-MAMBA method accurately captures the turning motion, and the predicted trajectory is highly consistent with the ground-truth trajectory in terms of both trajectory shape and endpoint position. This mainly benefits from two aspects. On the one hand, the de-occlusion completion module recovers motion-state information for occluded frames, providing the prediction module with continuous, complete historical-motion context, thereby preventing missing information from being directly propagated to the prediction stage. On the other hand, the Temporal Bi-Mamba module models long-term temporal dependencies in the completed trajectory in both the forward and backward directions, enabling the model to combine motion trends before and after the occlusion to make more accurate predictions. The qualitative results corroborate the quantitative conclusions in Table 3 and Table 4: the proposed method achieves the lowest errors across all occlusion ratios, indicating that it not only reduces prediction error but also generates trajectories that better conform to pedestrian-motion patterns.

#### 4.4.3. Failure Case Analysis

**Figure 6:**
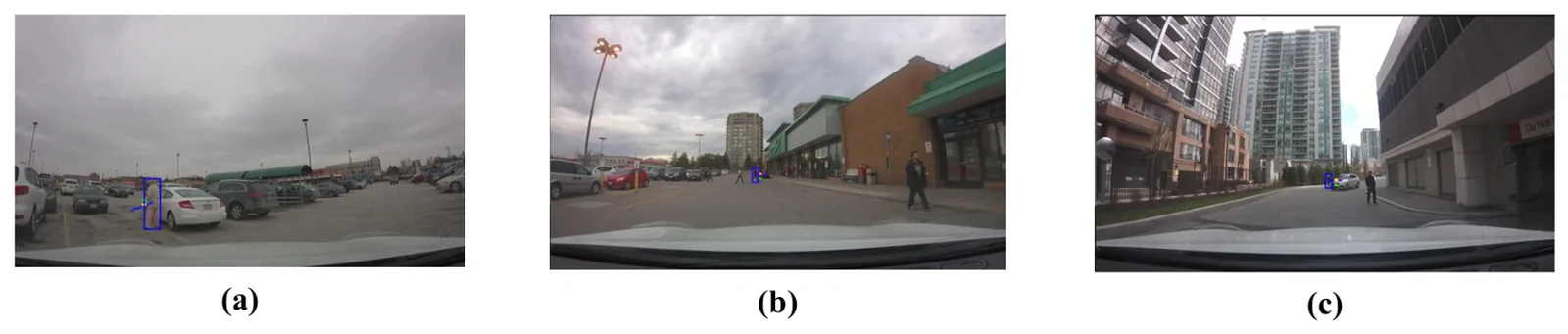
Typical failure case in occluded-trajectory recovery. Blue solid line: real trajectory; red region: occluded segment; yellow curve: mean interpolation baseline; green curve: RCN recovery result of this work. From left to right: (**a**) abrupt turns, (**b**) dense interactions, and (**c**) long occlusions.

**Figure 7:**
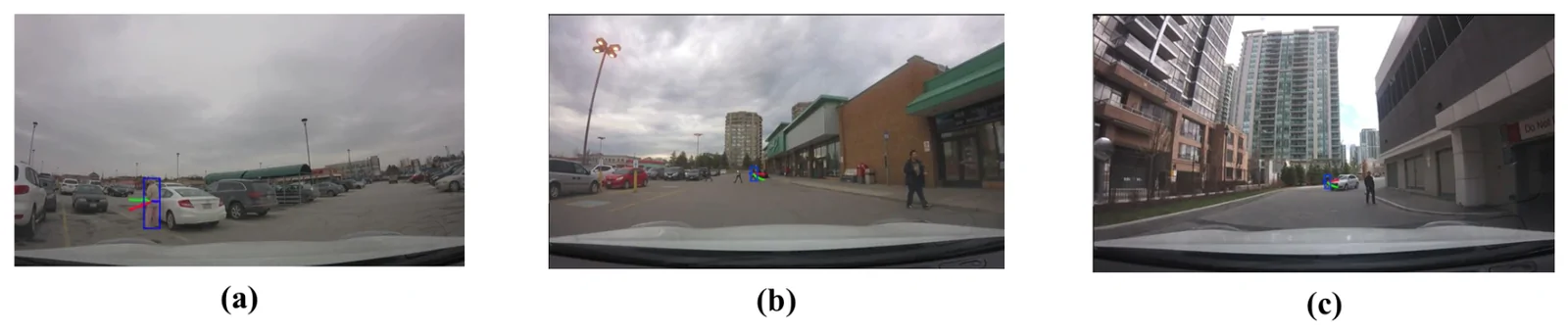
Typical failure case in future-trajectory prediction. Blue: historical observations; red: ground-truth future trajectories; green: model predictions. From left to right: (**a**) abrupt turns, (**b**) dense interactions, and (**c**) long occlusions.

To further improve the transparency and rigor of the evaluation, we provide an analysis of successful cases in addition to failure cases and boundary scenarios. Figure 6 and Figure 7 illustrate typical failure cases of the proposed method for occluded-trajectory recovery and future-trajectory prediction, respectively, covering three boundary scenarios: sudden turning, dense interaction, and long-term continuous occlusion.

In the sudden turning scenario (Figure 6a/Figure 7a), the pedestrian undergoes a significant direction change within the occluded segment, and the visible historical frames lack sufficient evidence of the turning motion, making it difficult for the recovery stage to accurately reconstruct this abrupt motion change; the predicted trajectory consequently exhibits a deviation near the turning point.

In the dense interaction scenario (Figure 6b/Figure 7b), multiple pedestrians move in close proximity in parallel or crossing patterns. Since the proposed method currently only utilizes the historical-trajectory information of a single pedestrian and does not explicitly model interaction relationships among pedestrians, the predicted trajectory can hardly accurately reflect motion adjustments arising from interaction effects. This is also one of the future research directions pointed out in Section 5.

In the long-term continuous occlusion scenario (Figure 6c/Figure 7c), the occluded segment lasts 5–6 frames, and the continuous missingness results in severely insufficient available motion information, leading to large errors in both the recovered and predicted trajectories. This is consistent with the quantitative conclusion in Table 3 and Table 4, which shows that the error under continuous occlusion worsens as the occlusion ratio increases.

Overall, the failure cases mainly concentrate on extreme scenarios such as abrupt motion-state changes, dense interaction, and long-term continuous occlusion, which is consistent with the quantitative trends in Table 3 and Table 4. In contrast, under conventional occlusion scenarios, the proposed method exhibits stable recovery and prediction capability. The aforementioned failure patterns also provide a clear direction for the subsequent introduction of multimodal information, interaction modeling, and real occlusion data.

### 4.5. Computational Complexity Analysis

To evaluate the feasibility of the proposed method for practical deployment in autonomous driving, this section compares RCN-MAMBA with typical LSTM- and Transformer-based prediction methods in terms of theoretical complexity and measured overhead.

#### 4.5.1. Theoretical Complexity Analysis

Let the historical sequence length be *T*, and the feature dimension be *d*. The computational complexity of LSTM per time step is $O(d^{2})$, totaling $O(T\cdot d^{2})$ over *T* time steps. However, its recursive dependency prevents parallelization along the temporal direction, and the sequence length constrains the actual inference latency. The self-attention complexity of Transformer is $O(T^{2}\cdot d)$, which grows quadratically with the sequence length and incurs significant overhead in long-sequence scenarios. Mamba-style state-space models achieve linear complexity of $O(T\cdot d^{2})$ through selective scanning while supporting efficient sequence scanning.

The computational cost of the proposed RCN-MAMBA mainly consists of three parts: the BiLSTM residual network in the de-occlusion stage, the explicit motion-state encoding and multi-scale temporal convolution (MTC) in the prediction stage, and the Temporal Bi-Mamba (TMB). Among them, the occluded segment length fed into the BiLSTM residual network is much shorter than the full sequence length and the feature dimension is small, so its complexity is $O(T\cdot d_{c}^{2})$; MTC adopts three temporal convolution branches with fixed kernel sizes, with a complexity of $O(T\cdot d^{2})$; and TMB consists of a forward and a backward Mamba module, with a complexity of $O(2T\cdot d^{2})$. Therefore, the overall time complexity of the proposed method is $O(T\cdot d^{2})$, which is linear in the sequence length, and lower than the $O(T^{2}\cdot d)$ of Transformer; it has the same order as LSTM but avoids the sequential delay caused by recursion through linear scanning, and the two directional Mamba modules can be executed in parallel.

#### 4.5.2. Empirical Comparison

To validate the theoretical analysis and evaluate the practical deployment overhead, we measure the number of parameters, per-trajectory inference time, and FLOPs for each method on an RTX 3060 GPU with the same input length ($T_{h}=15$); the results are shown in Table 12. Specifically, the number of parameters is obtained from the model’s static structure; the inference time is averaged over 1000 repeated measurements with a batch size of 1 on a single GPU; and the FLOPs are computed for a single forward pass.

As can be seen from Table 12, (1) in terms of the number of parameters, the proposed method has 2.5 M parameters, which is about 30.6% lower than that of Transformer (3.6 M) and is in the same order of magnitude as LSTM (1.2 M) and Trajectory Mamba (1.9 M). (2) In terms of inference time, the inference time of the proposed method for a single trajectory is 4.2 ms, far below the real-time budget of 33 ms that corresponds to the 30Hz acquisition period of the onboard camera, and is 12.5% lower than that of Transformer (4.8 ms), while including the complete pipeline of de-occlusion completion and future prediction. (3) In terms of computational cost, the FLOPs of the proposed method are 0.16 G, comparable to Mamba-style methods and significantly lower than those of Transformer and MS-TIP. These results demonstrate that the proposed method maintains near-linear computational complexity and real-time inference capability while achieving the best prediction accuracy, and can satisfy the deployment requirements of autonomous-driving scenarios.

## 5. Conclusions

### 5.1. Work Summary

This paper studies the problem of pedestrian-future-trajectory prediction under occlusion. With the aim of looking at the issue of pedestrians’ historical trajectories being easily affected by occlusion in real traffic scenes, this paper proposes an overall method based on de-occlusion completion and a MAMBA-based future-trajectory-prediction model. First, this paper clarifies the need for research on pedestrian-future-trajectory prediction under occlusion. Occlusion destroys the continuity of historical trajectories, making it difficult for prediction models to accurately obtain the true motion trends. Therefore, this paper adopts an overall framework of “de-occlusion first, prediction second”, modeling historical-trajectory recovery and future-trajectory prediction as a continuous pipeline. Second, in terms of de-occlusion, this paper proposes a physics-aware residual completion model. Taking the interpolated trajectory as the initial estimate, the model constructs motion features such as position, velocity, acceleration, distances to the left and right boundaries, and the occlusion mask, predicts the acceleration corrections and confidence through the residual-correction network, and recovers the occluded trajectory via motion integration. This method alleviates the problems that pure interpolation is overly smooth and that it is difficult to recover complex motion changes. Finally, in terms of future-trajectory prediction, this paper enhances the expression of dynamic information through explicit motion-state encoding, extracts local motion patterns at different temporal scales through multi-scale temporal convolution, and models bidirectional long-term temporal dependencies through Temporal Bi-Mamba. Compared with the baseline, the complete method of this paper achieves varying degrees of reduction in terms of the averages of ADE (0.5 s), ADE (1.0 s), ADE (1.5 s), FDE, C-ADE (0.5 s), C-ADE (1.0 s), C-ADE (1.5 s), and C-FDE across the six occlusion ratios, indicating that the proposed method still maintains good stability and robustness under high-occlusion conditions.

### 5.2. Discussion

The experimental results demonstrate that the proposed two-stage framework of de-occlusion first, prediction second, significantly outperforms the Baseline that directly predicts from the occluded trajectories under occlusion, and the advantage expands as the occlusion ratio increases. The ablation study further reveals the contribution order of each module: physics-aware residual completion contributes the most, followed by explicit motion-state encoding, multi-scale temporal convolution, and Temporal Bi-Mamba. This indicates that in occlusion scenarios, the quality of historical-trajectory recovery plays a decisive role in final prediction performance; meanwhile, motion-state encoding and bidirectional temporal modeling can further exploit motion patterns in the completed trajectory, and the two stages exhibit a clear complementary relationship.

The failure case analysis in Section 4.4.3 shows that the applicability boundary of the proposed method primarily depends on three factors: the degree of abrupt motion change within the occluded segment, the amount of missing information, and the complexity of scene interactions. When the pedestrian makes a sudden turn within the occluded segment, the visible historical information is insufficient to provide evidence of the turning motion, and both recovery and prediction tend to deviate; when the continuous occluded segment is too long, the available motion information is severely insufficient, the errors increase significantly, and in extreme cases the correction process may even introduce additional deviations; and when there are dense pedestrian interactions in the scene, since the proposed method only models a single pedestrian, the prediction can hardly reflect the motion adjustments caused by interactions. In contrast, under conventional occlusion conditions, the proposed method can stably recover trajectories and maintain high prediction accuracy. The aforementioned boundary conditions are consistent with the quantitative trends in Table 3 and Table 4 and indicate the direction of improvement for future research.

This paper conducts a systematic evaluation of the ego-vehicle-view datasets JAAD and PIE. Overall, the two datasets exhibit consistent patterns: the proposed method achieves the lowest errors under all occlusion ratios and both occlusion modes, the contribution order of each module is consistent, and continuous occlusion is always more challenging than random occlusion, indicating that the improvement of the proposed method has consistent generalizability across different datasets rather than relying on specific data distributions.

Compared with existing methods, this paper makes three unique contributions. First, compared with occlusion-aware imputation-prediction methods such as MS-TIP, the proposed method explicitly introduces physics-aware motion features and acceleration-domain residual correction in the completion stage, and guarantees the kinematic continuity of the completed trajectory in both position and velocity through motion integration, whereas existing imputation methods mainly reconstruct missing values based on statistical or attention mechanisms without explicit constraints on pedestrian-motion patterns. The comparative experiments under occlusion show that the proposed method achieves lower prediction errors. Second, compared with the existing prediction methods in Table 1 that use complete historical trajectories, these methods degrade significantly under occluded inputs, whereas the proposed method jointly models trajectory recovery and prediction, enabling the predictor to utilize the completed historical information. Third, unlike Mamba-based methods such as Trajectory Mamba that directly model complete trajectories, the proposed Temporal Bi-Mamba is designed for completed, noisy trajectories and, combined with explicit motion-state encoding and multi-scale temporal convolution, can more robustly model long-term temporal dependencies in occlusion-recovered sequences. In addition, this paper systematically evaluates both random and continuous occlusion modes, covering two types of real missingness causes, namely detector misses and structural occlusion, and demonstrates that continuous occlusion is a more challenging setting, providing a more complete evaluation benchmark for subsequent occlusion-aware prediction research.

### 5.3. Limitations

Although the proposed method has been improved for the task of pedestrian-future-trajectory prediction under occlusion, there is still room for further research. The occlusion scenarios in this paper are primarily generated using artificially synthesized occlusion masks. Although this setting can systematically simulate two typical missingness causes, namely detector misses and structural occlusion, it still differs essentially from occlusions in real road scenes: real occlusion is usually accompanied by partial visibility, cross-view switching, illumination changes, and the gradual nature of occlusion boundaries, and the distribution of real occlusion patterns is difficult to preset. In particular, when a moving object occludes a pedestrian, the occluded region changes dynamically with the occluding object’s motion, and such dynamic occlusion is difficult to fully simulate using static synthetic masks. The generalization performance of the proposed method on real occlusion data remains to be verified. Future work will study completion and prediction methods for real occlusion scenarios.

The proposed method uses only the historical-trajectory information of a single pedestrian for completion and prediction and does not explicitly model interactions between the pedestrian and other traffic participants. In dense interaction scenarios, multiple pedestrians move in proximity in parallel or crossing patterns, and the predicted trajectories of the proposed method can hardly accurately reflect motion adjustments due to interaction effects, leading to increased prediction errors. This indicates that the current method lacks sufficient modeling capability for complex multi-pedestrian-interaction scenarios and is primarily applicable to sparse-interaction scenarios. Future work will introduce group-interaction relationships, pedestrian–vehicle interaction relationships, and environmental-constraint information to improve the model’s predictive capability in complex multi-pedestrian-interaction scenarios.

The proposed method depends on the quality of the upstream detector. The trajectory input comes directly from the bounding box coordinates output by the detector, and detection quality issues such as detector misses, false detections, bounding-box jitter, and noise are propagated to the completion and prediction stages, affecting final prediction accuracy. This paper simulates trajectory missingness due to detector misses using synthetic occlusion masks, but does not explicitly model more complex detection-degradation cases, such as bounding-box noise and target-identity switches. The impact of these detection-quality issues on trajectory completion and prediction performance under occlusion still requires further investigation. Future work will explore joint modeling of detection confidence and trajectory completion to enhance the method’s robustness when processing low-quality detection inputs.

The proposed method primarily performs completion and prediction based on trajectory coordinates and does not utilize multimodal information such as image semantics, road structure, traffic signals, or vehicle speed. When historical information is severely missing, the motion evidence available from single-coordinate input is limited, making it difficult to accurately recover abrupt changes in motion during occlusion. Meanwhile, deterministic single-step decoding cannot explicitly model the multimodal distribution of future trajectories. Future work will integrate multimodal information to improve the model’s understanding of complex traffic scenes and study more lightweight Mamba structures, adaptive temporal-fusion mechanisms, and multimodal prediction heads to improve prediction diversity while reducing computational complexity.

## Figures and Tables

**Table 1:** Model architecture and training hyperparameter settings.

Category	Hyperparameter	Value
Model structure	Feature embedding dimension *d*	128
	De-occlusion BiLSTM hidden dimension	64
	MTC convolution kernel widths	1, 2, 4
	Number of TMB layers *N*	2
	Mamba state dimension $d_{\mathrm{state}}$	16
	Mamba expansion factor $d_{\mathrm{expand}}$	2
	Mamba convolution kernel width $d_{\mathrm{conv}}$	4
	Time-step rank dt_rank	8
	Normalization	RMSNorm
	Activation function	SiLU
	Decoder hidden dimension	128
	Dropout	0.1
Training	Optimizer	Adam
	Learning rate	$1\times 10^{-3}$
	Learning rate scheduler	CosineAnnealingLR
	Weight decay	$1\times 10^{-4}$
	Batch size	128
	Number of epochs	50
	Completion-loss weight $\gamma_{\mathrm{ms}}$	$1\times 10^{-3}$
	Prediction-loss weight $\lambda_{\mathrm{pred}}$	1.0

**Table 2:** Comparison of Future-Trajectory-Prediction Performance (Best Trajectory). The best results are highlighted in bold.

Method	JAAD					PIE				
	ADE0.5s	ADE1.0s	ADE1.5s	C-ADE	C-FDE	ADE0.5s	ADE1.0s	ADE1.5s	C-ADE	C-FDE
BiTrap-NP (2021) [46]	38	94	222	177	565	23	48	102	81	261
SGNet (2022) [47]	37	86	197	146	443	16	39	88	66	206
ABC+ (2022) [48]	40	89	189	145	409	16	38	87	65	191
ENCORE (2024) [27]	32	85	210	167	554	15	33	70	49	155
PQPCP (2024) [49]	29	66	147	113	322	14	32	67	47	127
GoalNet (2025) [50]	31	67	133	97	315	15	32	65	46	121
NIM-STGCN (2026) [51]	28	63	124	101	275	14	31	57	44	97
Ours	**25**	**57**	**112**	**86**	**238**	**13**	**28**	**52**	**38**	**84**

**Table 3:** Experimental results on the JAAD dataset under different occlusion ratios. The best results are highlighted in bold.

Occlusion Ratio	Occlusion Mode	Method	ADE0.5s	ADE1.0s	ADE1.5s	FDE	C-ADE	C-FDE
5%	Random/Continuous	Baseline	36.8	79.5	151.7	326.4	124.5	283.6
		Mean interpolation + SGNet	32.4	70.8	136.9	294.2	111.8	259.7
		MS-TIP	29.8	65.4	126.3	271.5	102.6	252.4
		RCN-MAMBA	**27.1**	**61.2**	**120.8**	**255.9**	**91.7**	**248.6**
10%	Random	Baseline	42.7	91.8	176.9	382.5	145.2	331.7
		Mean interpolation + SGNet	37.9	81.2	156.8	339.6	128.4	297.3
		MS-TIP	33.6	73.5	142.7	308.9	115.7	275.6
		RCN-MAMBA	**29.4**	**65.7**	**129.6**	**271.8**	**96.4**	**258.9**
	Continuous	Baseline	46.3	99.1	190.7	412.5	156.8	359.1
		Mean interpolation + SGNet	41.5	88.7	170.3	367.2	138.9	318.4
		MS-TIP	35.8	78.4	151.6	327.5	122.3	289.7
		RCN-MAMBA	**30.1**	**67.8**	**133.9**	**284.2**	**99.8**	**269.3**
15%	Random	Baseline	48.6	104.3	201.8	431.6	164.8	372.4
		Mean interpolation + SGNet	42.8	92.6	178.5	383.4	145.7	330.2
		MS-TIP	37.2	81.9	158.3	341.6	126.8	298.5
		RCN-MAMBA	**31.6**	**70.5**	**138.7**	**289.6**	**101.8**	**274.5**
	Continuous	Baseline	49.9	107.6	203.9	435.1	167.0	377.9
		Mean interpolation + SGNet	44.3	95.7	184.2	391.5	149.6	338.9
		MS-TIP	38.5	84.6	163.7	352.4	130.2	306.8
		RCN-MAMBA	**33.7**	**74.6**	**139.9**	**290.1**	**104.9**	**276.8**
20%	Random	Baseline	56.9	121.7	232.6	498.7	190.5	428.3
		Mean interpolation + SGNet	47.3	104.6	201.4	428.7	158.2	356.4
		MS-TIP	41.8	92.4	178.5	381.2	141.7	326.9
		RCN-MAMBA	**34.2**	**76.8**	**151.4**	**315.2**	**109.6**	**298.7**
	Continuous	Baseline	63.2	135.2	259.1	556.8	212.4	478.6
		Mean interpolation + SGNet	55.8	121.9	231.4	493.2	186.4	416.8
		MS-TIP	48.6	108.3	205.7	442.1	167.3	376.5
		RCN-MAMBA	**35.9**	**81.2**	**159.6**	**334.7**	**115.2**	**314.8**
30%	Random	Baseline	70.8	150.9	285.7	611.5	235.4	526.8
		Mean interpolation + SGNet	60.7	131.2	252.8	538.4	203.5	457.1
		MS-TIP	52.4	114.8	221.6	475.3	178.9	404.2
		RCN-MAMBA	**39.8**	**87.6**	**171.9**	**359.7**	**124.3**	**339.2**
	Continuous	Baseline	85.4	182.6	350.2	754.8	288.1	651.3
		Mean interpolation + SGNet	74.2	158.7	302.5	648.9	245.3	551.2
		MS-TIP	63.9	138.5	267.2	576.4	215.6	489.3
		RCN-MAMBA	**44.8**	**100.7**	**197.8**	**415.9**	**141.6**	**392.4**
40%	Random	Baseline	86.4	184.7	342.6	731.5	282.6	604.2
		Mean interpolation + SGNet	72.8	156.4	298.6	638.2	240.7	541.3
		MS-TIP	62.7	135.9	261.3	558.7	211.4	476.8
		RCN-MAMBA	**45.7**	**97.4**	**188.6**	**391.5**	**150.8**	**358.6**
	Continuous	Baseline	102.3	219.8	424.5	912.7	350.6	793.4
		Mean interpolation + SGNet	88.7	189.3	362.4	779.5	296.8	665.2
		MS-TIP	76.4	164.2	316.8	682.4	259.3	584.6
		RCN-MAMBA	**51.2**	**115.4**	**227.9**	**476.3**	**162.5**	**455.8**

**Table 4:** Experimental results from the PIE dataset under different occlusion ratios. The best results are highlighted in bold.

Occlusion Ratio	Occlusion Mode	Method	ADE0.5s	ADE1.0s	ADE1.5s	FDE	C-ADE	C-FDE
5%	Random/Continuous	Baseline	18.2	38.5	71.3	149.2	55.1	100.8
		Mean interpolation + SGNet	16.3	34.6	64.2	134.5	49.6	93.4
		MS-TIP	15.1	32.3	59.8	124.7	45.9	91.2
		RCN-MAMBA	**14.1**	**30.2**	**56.2**	**117.5**	**42.3**	**89.2**
10%	Random	Baseline	21.3	44.6	82.8	173.5	63.8	117.5
		Mean interpolation + SGNet	19.2	40.3	74.6	156.8	57.4	106.2
		MS-TIP	17.3	36.8	68.4	143.5	51.9	99.7
		RCN-MAMBA	**15.2**	**33.1**	**61.5**	**129.8**	**45.6**	**93.6**
	Continuous	Baseline	24.3	50.8	93.9	198.3	72.4	133.5
		Mean interpolation + SGNet	21.9	45.7	84.3	177.6	65.2	120.4
		MS-TIP	19.1	40.2	74.8	157.3	56.8	110.6
		RCN-MAMBA	**16.4**	**36.4**	**67.8**	**143.2**	**50.0**	**102.8**
15%	Random	Baseline	24.5	50.8	94.6	196.8	72.5	133.2
		Mean interpolation + SGNet	22.1	45.9	85.2	177.4	65.4	120.2
		MS-TIP	19.3	40.6	75.6	158.7	57.2	111.3
		RCN-MAMBA	**16.3**	**35.8**	**66.8**	**140.5**	**49.2**	**99.5**
	Continuous	Baseline	25.6	51.1	96.9	197.5	74.7	135.6
		Mean interpolation + SGNet	23.1	47.8	88.4	183.2	67.3	123.8
		MS-TIP	20.2	42.3	78.5	164.8	59.4	114.6
		RCN-MAMBA	**16.4**	**36.0**	**67.1**	**141.1**	**49.4**	**99.9**
20%	Random	Baseline	28.2	58.4	109.5	227.3	83.6	151.8
		Mean interpolation + SGNet	23.6	50.7	95.2	198.1	70.4	140.6
		MS-TIP	20.8	44.9	84.7	176.4	62.3	124.8
		RCN-MAMBA	**17.5**	**38.6**	**72.4**	**152.6**	**53.5**	**106.8**
	Continuous	Baseline	32.6	67.7	126.5	263.2	96.6	176.4
		Mean interpolation + SGNet	27.9	59.4	110.6	229.8	81.5	161.3
		MS-TIP	24.5	52.8	98.3	204.7	72.8	144.2
		RCN-MAMBA	**18.9**	**41.8**	**78.1**	**164.9**	**57.4**	**115.8**
30%	Random	Baseline	34.8	71.6	136.2	282.5	103.8	187.5
		Mean interpolation + SGNet	29.4	62.8	118.3	245.6	87.2	172.1
		MS-TIP	25.7	55.3	104.6	217.5	76.9	152.4
		RCN-MAMBA	**19.6**	**42.8**	**81.5**	**166.8**	**55.8**	**121.2**
	Continuous	Baseline	42.1	87.2	165.3	344.1	125.8	230.5
		Mean interpolation + SGNet	36.2	76.1	142.5	296.4	104.8	206.9
		MS-TIP	31.6	67.4	126.8	264.2	93.5	185.7
		RCN-MAMBA	**23.2**	**50.9**	**96.7**	**197.6**	**65.9**	**144.8**
40%	Random	Baseline	42.5	87.5	166.8	342.6	127.5	226.8
		Mean interpolation + SGNet	34.8	73.5	138.9	287.3	101.4	200.5
		MS-TIP	30.2	64.7	122.3	252.8	89.7	177.6
		RCN-MAMBA	**22.8**	**49.2**	**93.5**	**190.5**	**67.5**	**132.5**
	Continuous	Baseline	50.8	104.7	199.5	414.2	153.1	282.6
		Mean interpolation + SGNet	42.6	89.7	167.5	348.2	122.6	241.8
		MS-TIP	36.9	78.4	148.2	308.6	108.5	214.3
		RCN-MAMBA	**26.6**	**57.9**	**109.8**	**223.4**	**78.1**	**163.9**

**Table 5:** Robustness analysis of the de-occlusion module against missing data on the JAAD dataset. The best results are highlighted in bold.

Masked%	De-Occlusion	MAE	RMSE	ADE	FDE
5%	Mean	18.6	28.9	27.5	56.8
	Linear	18.2	28.3	27.0	55.6
	Spline	18.0	28.0	26.8	55.1
	Spline+Residual	13.6	21.8	26.9	44.2
	**PRC**	**13.4**	**21.6**	**26.8**	**43.7**
10%	Mean	24.7	38.5	35.6	74.2
	Linear	23.4	36.7	33.9	70.8
	Spline	22.9	35.9	33.2	69.4
	Spline+Residual	17.4	28.1	28.7	60.8
	**PRC**	**16.9**	**27.4**	**27.8**	**58.6**
15%	Mean	30.5	47.2	42.8	88.5
	Linear	28.5	44.3	40.3	83.4
	Spline	27.8	43.2	39.4	81.6
	Spline+Residual	20.5	33.0	33.4	70.5
	**PRC**	**19.7**	**31.9**	**31.9**	**67.2**
20%	Mean	37.2	56.8	51.6	106.4
	Linear	34.3	52.9	48.2	99.3
	Spline	33.4	51.5	47.1	97.1
	Spline+Residual	24.3	38.3	38.8	81.8
	**PRC**	**23.1**	**36.7**	**36.7**	**76.9**
30%	Mean	48.5	72.9	65.4	136.7
	Linear	44.1	66.7	60.3	125.9
	Spline	42.8	64.8	58.7	122.7
	Spline+Residual	30.6	47.1	45.6	97.4
	**PRC**	**28.9**	**44.8**	**42.9**	**91.3**
40%	Mean	61.7	91.6	82.7	172.5
	Linear	55.3	83.1	75.4	157.0
	Spline	53.6	80.6	73.2	152.8
	Spline+Residual	35.6	55.5	53.2	113.2
	**PRC**	**33.5**	**52.6**	**49.8**	**105.7**

**Table 6:** Robustness analysis of the de-occlusion module against missing data on the PIE dataset. The best results are highlighted in bold.

Masked%	De-Occlusion	MAE	RMSE	ADE	FDE
5%	Mean	9.1	14.2	13.6	28.2
	Linear	8.9	13.9	13.3	27.6
	Spline	8.7	13.6	13.1	27.1
	Spline+Residual	6.7	10.7	13.2	21.9
	**PRC**	**6.6**	**10.6**	**13.2**	**21.7**
10%	Mean	12.1	18.9	17.6	36.8
	Linear	11.5	18.0	16.8	35.2
	Spline	11.2	17.6	16.4	34.5
	Spline+Residual	8.5	13.9	14.2	29.9
	**PRC**	**8.3**	**13.5**	**13.8**	**29.0**
15%	Mean	14.9	23.2	21.2	43.8
	Linear	13.9	21.8	19.9	41.2
	Spline	13.5	21.2	19.4	40.3
	Spline+Residual	10.0	16.2	16.5	34.9
	**PRC**	**9.6**	**15.7**	**15.8**	**33.3**
20%	Mean	18.2	27.8	25.5	52.6
	Linear	16.7	25.7	23.6	48.9
	Spline	16.2	25.0	23.0	47.7
	Spline+Residual	11.9	18.8	19.2	40.2
	**PRC**	**11.3**	**18.0**	**18.2**	**38.0**
30%	Mean	23.7	35.7	32.4	67.6
	Linear	21.4	32.5	29.5	61.6
	Spline	20.7	31.6	28.7	60.0
	Spline+Residual	14.9	23.0	22.6	48.1
	**PRC**	**14.1**	**21.9**	**21.3**	**45.2**
40%	Mean	30.1	44.8	41.0	85.5
	Linear	26.7	40.2	36.9	77.1
	Spline	25.8	39.2	35.8	74.9
	Spline+Residual	17.4	27.2	26.4	56.3
	**PRC**	**16.4**	**25.8**	**24.7**	**52.3**

**Table 7:** Ablation Study Results on JAAD Dataset (40% Occlusion Ratio). The checkmark (✓) indicates that the corresponding module is enabled, while the cross (×) indicates it is disabled. The best results are highlighted in bold.

De-Occlusion	EME	MTC	TBM	ADE0.5	ADE1.0	ADE1.5	FDE	C-FDE
×	×	×	×	86.4	184.7	342.6	731.5	604.2
Mean	×	×	×	74.8	156.9	293.4	621.8	516.4
Mean + Residual	×	×	×	52.6	111.8	214.7	452.6	372.8
Mean + Residual	✓	×	×	46.3	99.4	191.6	405.2	334.7
Mean + Residual	✓	✓	×	39.8	84.7	165.2	346.5	286.4
Mean + Residual	✓	✓	✓	**30.7**	**68.5**	**132.4**	**278.9**	**238.6**

**Table 8:** Ablation Study Results on PIE Dataset (40% Occlusion Ratio). The checkmark (✓) indicates that the corresponding module is enabled, while the cross (×) indicates it is disabled. The best results are highlighted in bold.

De-Occlusion	EME	MTC	TBM	ADE0.5	ADE1.0	ADE1.5	FDE	C-FDE
×	×	×	×	44.9	90.7	159.1	348.3	213.3
Mean	×	×	×	38.9	77.1	136.2	296.1	182.3
Mean + Residual	×	×	×	27.4	54.9	99.7	215.5	131.6
Mean + Residual	✓	×	×	24.1	48.8	89.0	193.0	118.1
Mean + Residual	✓	✓	×	20.7	41.6	76.7	165.0	101.1
Mean + Residual	✓	✓	✓	**16.0**	**33.7**	**61.5**	**132.8**	**84.2**

**Table 9:** Statistical significance of the incremental contribution of each module in the ablation study on the JAAD dataset (40% occlusion).

Comparison	ADE0.5	ADE1.0	ADE1.5	FDE	C-FDE
+Mean interpolation vs. Baseline	−11.6(−13.1,−10.1), p<0.001	−27.8(−31.1,−24.5), p<0.001	−49.2(−55.2,−43.2), p<0.001	−109.7(−122.6,−96.8), p<0.001	−87.8(−98.4,−77.2), p<0.001
+Residual correction vs. +Mean	−22.2(−23.5,−20.9), p<0.001	−45.1(−47.9,−42.3), p<0.001	−78.7(−83.9,−73.5), p<0.001	−169.2(−180.1,−158.3), p<0.001	−143.6(−152.7,−134.5), p<0.001
+EME vs. +Residual correction	−6.3(−7.2,−5.4), p<0.001	−12.4(−14.4,−10.4), p<0.001	−23.1(−26.9,−19.3), p<0.001	−47.4(−55.4,−39.4), p<0.001	−38.1(−44.7,−31.5), p<0.001
+MTC vs. +EME	−6.5(−7.3,−5.7), p<0.001	−14.7(−16.4,−13.0), p<0.001	−26.4(−29.8,−23.0), p<0.001	−58.7(−65.8,−51.6), p<0.001	−48.3(−54.2,−42.4), p<0.001
+TBM vs. +MTC	−9.1(−9.8,−8.4), p<0.001	−16.2(−17.7,−14.7), p<0.001	−32.8(−35.7,−29.9), p<0.001	−67.6(−73.7,−61.5), p<0.001	−47.8(−52.8,−42.8), p<0.001

**Table 10:** Statistical significance of the incremental contribution of each module in the ablation study on the PIE dataset (40% occlusion).

Comparison	ADE0.5	ADE1.0	ADE1.5	FDE	C-FDE
+Mean interpolation vs. Baseline	−6.0(−6.7,−5.3), p<0.001	−13.6(−15.1,−12.1), p<0.001	−22.9(−25.5,−20.3), p<0.001	−52.2(−57.8,−46.6), p<0.001	−31.0(−34.4,−27.6), p<0.001
+Residual correction vs. +Mean	−11.5(−12.1,−10.9), p<0.001	−22.2(−23.4,−21.0), p<0.001	−36.5(−38.7,−34.3), p<0.001	−80.6(−85.4,−75.8), p<0.001	−50.7(−53.6,−47.8), p<0.001
+EME vs. +Residual correction	−3.3(−3.7,−2.9), p<0.001	−6.1(−7.0,−5.2), p<0.001	−10.7(−12.3,−9.1), p<0.001	−22.5(−26.0,−19.0), p<0.001	−13.5(−15.6,−11.4), p<0.001
+MTC vs. +EME	−3.4(−3.8,−3.0), p<0.001	−7.2(−8.0,−6.4), p<0.001	−12.3(−13.7,−10.9), p<0.001	−28.0(−31.1,−24.9), p<0.001	−17.0(−18.9,−15.1), p<0.001
+TBM vs. +MTC	−4.7(−5.0,−4.4), p<0.001	−7.9(−8.6,−7.2), p<0.001	−15.2(−16.4,−14.0), p<0.001	−32.2(−34.9,−29.5), p<0.001	−16.9(−18.5,−15.3), p<0.001

**Table 11:** Loss-term ablation results on the JAAD dataset at 40% occlusion.

Setting	ADE0.5s	ADE1.0s	ADE1.5s	FDE
Full loss	30.7	68.5	132.4	278.9
w/o $L_{\mathrm{vel}}$	32.1	72.3	139.5	292.8
w/o $L_{\mathrm{conf}}$	31.4	70.2	136.2	285.6
w/o $L_{\mathrm{sparse}}$	30.9	69.1	133.2	281.4

**Table 12:** Comparison of computational complexity of different methods (RTX 3060, $T_{h}=15$).

Model	Params (M)	Inference Time (ms)	FLOPs (G)
LSTM	1.2	2.9	0.09
Transformer	3.6	4.8	0.23
SGNet	2.9	5.4	0.20
MS-TIP	2.2	6.7	0.26
Trajectory Mamba	1.9	3.5	0.13
RCN-MAMBA (Ours)	2.5	4.2	0.16

## Data Availability

Data Availability Statement: The data used in this study are openly available in the Joint Attention in Autonomous Driving (JAAD) dataset [44] and the Pedestrian Intention Estimation (PIE) dataset [45].
